# Structurally Informed Mutagenesis of a Stereochemically Promiscuous Aldolase Produces Mutants That Catalyze the Diastereoselective Syntheses of All Four Stereoisomers of 3-Deoxy-hexulosonic Acid

**DOI:** 10.1021/acscatal.2c03285

**Published:** 2022-09-06

**Authors:** Sylvain F. Royer, Xuan Gao, Robin R. Groleau, Marc W. van der Kamp, Steven D. Bull, Michael J. Danson, Susan J. Crennell

**Affiliations:** † Department of Biology and Biochemistry, University of Bath, Bath BA2 7AY, U.K.; ‡ School of Biochemistry, University of Bristol, University Walk, Bristol BS8 1TD, U.K.; § Department of Chemistry, University of Bath, Bath BA2 7AY, U.K.

**Keywords:** enzyme engineering, stereospecificity, aldolase, crystal structures, *Sulfolobus solfataricus*, carbon−carbon bond formation

## Abstract

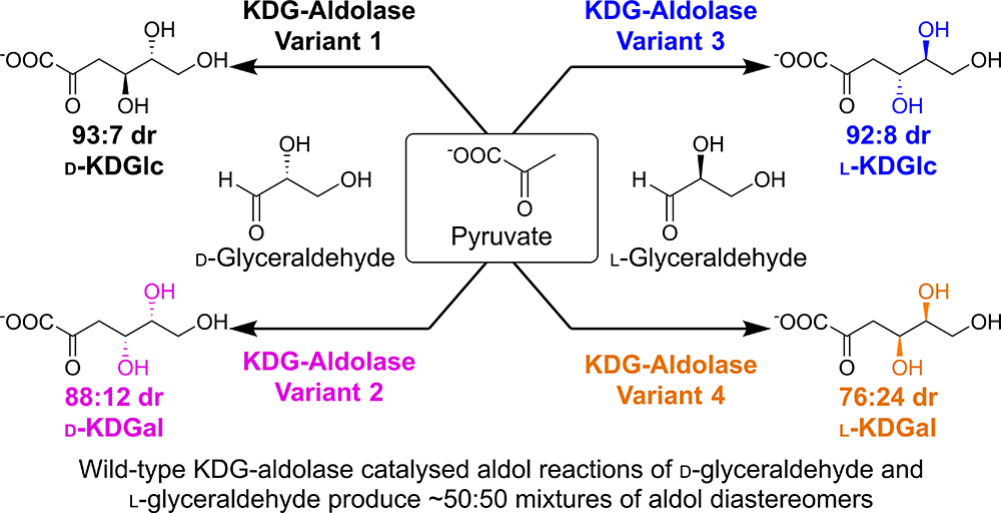

A 2-keto-3-deoxygluconate aldolase from the hyperthermophile *Sulfolobus solfataricus* catalyzes the nonstereoselective aldol reaction of pyruvate and d-glyceraldehyde to produce 2-keto-3-deoxygluconate (d-KDGlc) and 2-keto-3-deoxy-d-galactonate (d-KDGal). Previous investigations into curing the stereochemical promiscuity of this hyperstable aldolase used high-resolution structures of the aldolase bound to d-KDGlc or d-KDGal to identify critical amino acids involved in substrate binding for mutation. This structure-guided approach enabled mutant variants to be created that could stereoselectively catalyze the aldol reaction of pyruvate and natural d-glyceraldehyde to selectively afford d-KDGlc or d-KDGal. Here we describe the creation of two further mutants of this *Sulfolobus* aldolase that can be used to catalyze aldol reactions between pyruvate and non-natural l-glyceraldehyde to enable the diastereoselective synthesis of l-KDGlc and l-KDGal. High-resolution crystal structures of all four variant aldolases have been determined (both unliganded and liganded), including Variant 1 with d-KDGlc, Variant 2 with pyruvate, Variant 3 with l-KDGlc, and Variant 4 with l-KDGal. These structures have enabled us to rationalize the observed changes in diastereoselectivities in these variant-catalyzed aldol reactions at a molecular level. Interestingly, the active site of Variant 4 was found to be sufficiently flexible to enable catalytically important amino acids to be replaced while still retaining sufficient enzymic activity to enable production of l-KDGal.

## Introduction

Aldolases are a class of carbon–carbon bond-forming enzymes that catalyze the reversible aldol reaction between a carbonyl donor and an aldehyde acceptor to afford chiral aldol products containing up to two stereocentres.^1,2^ They have been widely used as biocatalysts for the stereoselective transformation of protecting-group-free substrates into sugar derivatives, enzyme inhibitors, metabolites, heterocycles, and drug precursors, all under environmentally friendly aqueous conditions.^3−6^ Aldolases are normally specific for their natural nucleophilic donors, which can significantly restrict the range of aldol products that can be produced.^7^ However, many aldolases display greater promiscuity toward their electrophilic aldehyde substrates, which allows significant structural diversity to be incorporated into their aldol products.^8^ Most aldolases produce aldol products as single stereoisomers containing stereocenters with a defined configuration, meaning that there is often no way of accessing stereoisomeric aldol products that are potentially desirable.^9^ This has led to molecular biology and protein engineering techniques being developed to improve the specificity and/or stereoselectivity profiles of aldolases toward their donor and acceptor substrates.^10^ Site-directed mutagenesis, gene shuffling, and directed evolution approaches have been used to evolve the reactivity profiles of aldolases toward non-natural donors and aldehyde electrophiles, with the aim of using mutant biocatalysts to produce chiral aldol products from non-native substrates.^11^ Similar success has also been achieved in improving the stereoselectivities of aldolase-catalyzed reactions using mutant aldolases that exhibit improved or inverted enantioselectivities/diastereoselectivities in their aldol reactions.^12−14^ However, many of these approaches require expensive and time-consuming screening of large libraries of mutant enzymes to achieve the desired changes in their reactivity profiles. Alternative approaches to modify specificity profiles have also been explored, based on structurally informed site-directed mutagenesis of key amino acid residues in the aldolase active site.^15^ This approach requires an intimate knowledge of the catalytic mechanism of the aldolase and an understanding of how donor/acceptor substrates (or aldol products) bind to the aldolase active site. This structural information can then be used to target key amino acid “hot spots” for replacement to produce mutants with new or improved selectivity profiles.

Pyruvate (and other 2-oxo acid) aldolases are an attractive class of carbon–carbon bond-forming enzymes that catalyze the aldol reaction of inexpensive pyruvate with a range of aldehydes to afford synthetically versatile chiral γ-hydroxy-α-keto acids that contain four contiguous carbon atoms at different oxidation states.^16^ Screening of aldolase collections has identified aldolases with complementary stereoselectivities for the same aldol reaction, thus providing biocatalysts for the stereodivergent syntheses of enantiomeric aldol products.^17^ Extensive protein engineering studies have been carried out to widen the substrate specificity profiles of pyruvate aldolases, with the aim of improving/inverting their stereoselectivities toward their aldehyde substrates.^16f,18^ An alternative approach to inverting the stereochemical preference of a stereoselective aldolase is to mutate an aldolase that catalyzes aldol reactions with poor levels of stereocontrol to produce variants that produce stereoisomeric aldol products with better levels of stereocontrol. This stereodivergent approach is a potentially efficient way of generating complementary mutant aldolase activities because the wild-type enzyme already displays good catalytic activity for production of each aldol stereoisomer. The challenge in this approach is to increase the catalytic activity of the aldolase for formation of the desired aldol stereoisomer and/or decrease the rate of formation of the unwanted aldol stereoisomer(s).

**Figure 1:**
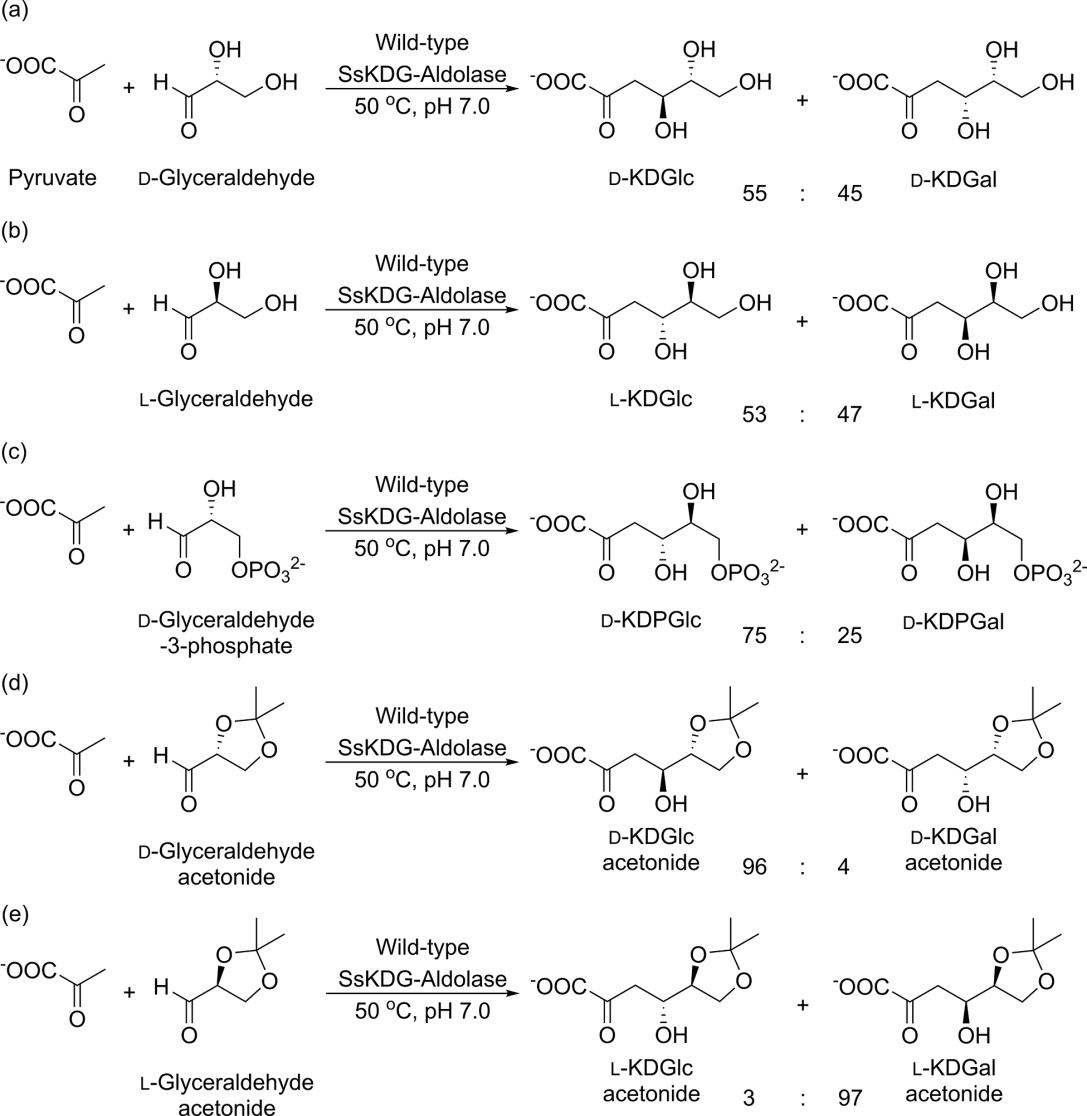
Wild-type SsKDG-aldolase catalyzed reactions of pyruvate with (a) d-glyceraldehyde; (b) l-glyceraldehyde; (c) d-glyceraldehyde-3-phosphate; (d) d-glyceraldehyde acetonide; and (e) l-glyceraldehyde acetonide. All aldol products are shown in their open-chain forms for clarity.

*Sulfolobus solfataricus* is a hyperthermophilic archaeon that grows optimally at 80 °C and metabolizes sugars via a nonphosphorylative variant of the Entner–Doudoroff pathway, with a series of stereochemically promiscuous enzymes used to metabolize the four most-common sugars in Nature, namely, d-glucose, d-galactose, d-ribose, and l-arabinose.^19,20^ The aldolase from this pathway [SsKDG-aldolase]^21^ catalyzes retro-aldol cleavage of both 2-keto-3-deoxy-d-gluconate and 2-keto-3-deoxy-d-galactonate into pyruvate and glyceraldehyde, as well as catalyzing cleavage of 2-keto-3-deoxy-d-xylonate and 2-keto-3-deoxy-l-arabinonate to pyruvate and glycolaldehyde.^22^ This aldolase is thermally stable, accepts a wide range of non-natural aldehyde substrates (e.g., the enantiomers of glyceraldehyde, threose, and erythrose), is tolerant to a range of cosolvents, and its gene has been efficiently expressed in *Escherichia coli*.^23^ However, its aldol reactions often proceed with poor levels of diastereocontrol, with wild-type SsKDG-aldolase catalyzing the aldol reaction of pyruvate and its natural d-glyceraldehyde substrate to afford a mixture of 2-keto-3-deoxy-d-gluconate (d-KDGlc) and 2-keto-3-deoxy-d-galactonate (d-KDGal) in a 55:45 diastereomeric ratio (dr) (Figure 1a).^24^ Conversely, the broad specificity profile of this aldolase means that it can also be used to catalyze aldol reaction of pyruvate with non-natural l-glyceraldehyde, affording a complementary 53:47 mixture of 2-keto-3-deoxy-l-gluconate (l-KDGlc) and 2-keto-3-deoxy-l-galactonate (l-KDGal) (Figure 1b).^24^

Although demonstrating good catalytic activity toward a range of aldehyde substrates, the lack of stereocontrol in these SsKDG-aldolase-catalyzed reactions is potentially problematic from a biocatalytic perspective because these aldol reactions produce mixtures of water-soluble diastereomeric aldol products that are difficult to separate. However, access to X-ray crystal structures of the wild-type aldolase bound to substrates and aldol products^25^ provided us with the opportunity to develop strategies to “cure” the aldolase’s inherent stereochemical promiscuity toward its natural d-glyceraldehyde substrate. Initial attempts to improve diastereocontrol in these aldolase reactions used a substrate-based strategy inspired by Nature, where charged phosphorylated substrates are often used to control binding affinities and selectivities in enzyme-catalyzed reactions. Therefore, it was found that the SsKDG-aldolase catalyzes the reaction of d-glyceraldehyde-3-phosphate with pyruvate^26,27^ to afford a mixture of d-KDPGlc and d-KDPGal in an improved 3:1 dr (Figure 1c) (S. F. Royer, unpublished data). Subsequent evaluation of aldolase crystal structures containing d-KDGlc and d-KDGal led us to consider using a more rigid cyclic d-glyceraldehyde acetonide substrate, which resulted in a highly diastereoselective aldol reaction that produced d-KDGlc and d-KDGal in 96:4 dr (Figure 1d).^24^ Interestingly, use of l-glyceraldehyde acetonide as a substrate resulted in an aldol reaction with opposing diastereoselectivity, affording a mixture of l-KDGlc and l-KDGal in 3:97 dr, thus indicating that the facial selectivity of both these aldol reactions occurs under enzymatic control (Figure 1e).^24,28^

**Figure 2:**
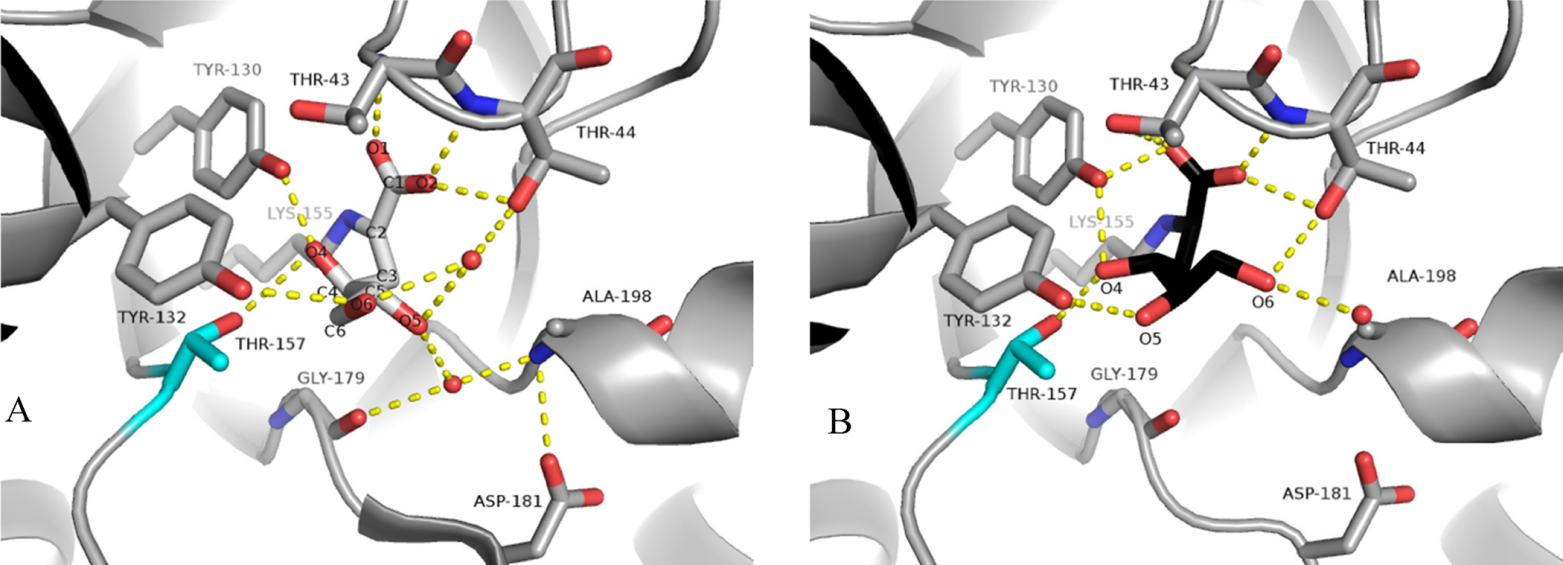
Active site of wild-type SsKDG-aldolase in complex with (A) d-KDGlc (PDB code: 1W3N, white bonds); and (B) d-KDGal (PDB code: 1W3T, black bonds) bound in the active site. The side chains of amino acids that interact with both aldol products are shown with hydrogen bonds indicated as yellow dotted lines. Bridging water molecules involved in key hydrogen bonding bridging are shown as red spheres.

**Figure 3:**
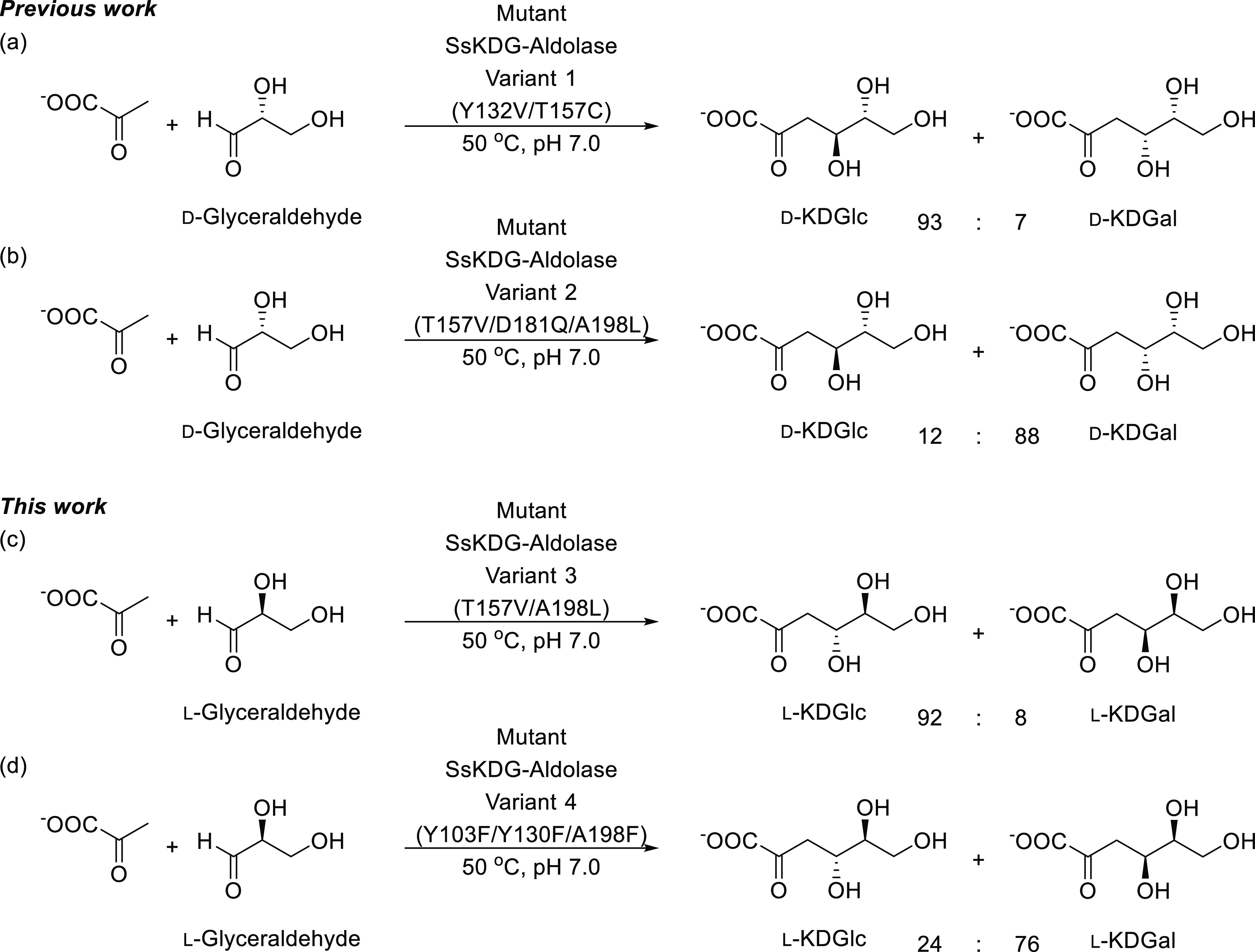
(a) Mutant SsKDG-aldolase Variant 1 for the stereoselective synthesis of d-KDGlc. (b) Mutant SsKDG-aldolase Variant 2 for the stereoselective synthesis of d-KDGal. (c) Mutant SsKDG-aldolase Variant 3 for the stereoselective synthesis of l-KDGlc. (d) Mutant SsKDG-aldolase Variant 4 for the stereoselective synthesis of l-KDGal. All aldol products are drawn in their open-chain forms for clarity.

We then employed X-ray crystal structures of the wild-type SsKDG-aldolase bound to d-KDGlc and d-KDGal (Figure 2) to identify a series of active-site amino acids for mutation that enabled stereocomplementary mutants to be identified, which were then used to selectively transform natural d-glyceraldehyde into d-KDGlc and d-KDGal, respectively. Therefore, a double mutant (Y132V/T157C; Variant 1) was identified that increased the proportion of d-KDGlc (over d-KDGal) from 55:45 to 93:7 dr (Figure 3a), while a triple mutant (T157V/D181Q/A198L; Variant 2) was created that increased the proportion of d-KDGal (over d-KDGlc) from 45:55 to 88:12 dr (Figure 3b).^29^

In this current study, we now report X-ray crystal structures of Variants 1 and 2 and Variant 1 bound to d-KDGlc that have enabled us to rationalize changes in the diastereoselectivity profiles of the aldol reactions of both these mutants at a molecular level. Furthermore, we also now report construction of two new SsKDG-aldolase variants that can be used to selectively transform non-natural l-glyceraldehyde into l-KDGlc or l-KDGal with good levels of diastereocontrol. This was achieved using structurally guided mutation approaches to create a double mutant (T157V/A198L; Variant 3) that increases the proportion of l-KDGlc produced from 53:47 to 92:8 dr (Figure 3c), while creation of a triple mutant (Y103F/Y130F/A198F; Variant 4) increased the amount of l-KDGal epimer produced from 43:57 to 76:24 dr (Figure 3d). Once again, these improvements in diastereocontrol have been rationalized at a molecular level by examining changes in the X-ray crystal structures of these new mutants complexed to l-KDGlc (Variant 3) and l-KDGal (Variant 4) in comparison with wild-type SsKDG-aldolase structures.

## Methods and Materials

### Bacterial Strains and Plasmids

*Escherichia coli* BL21 (DE3) competent cells were obtained from Stratagene, U.K., and used in conjunction with the pET-3a expression vector as described previously.^23^

### Creation and Purification of Site-Directed Mutants

Creation of Variant 1 (Y132V/T157C) and Variant 2 (T157V/D181Q/A198L) by site-directed mutagenesis has been reported previously.^29^ In the current study, two further mutant aldolases [Variant 3 (T157V/A198L) and Variant 4 (Y103F/Y130F/A198F)] were similarly created using a QuikChange II PCR kit obtained from Stratagene. Plasmids carrying the mutant genes were transformed into and expressed in *E. coli* BL21 (DE3), with the variant aldolases then purified from cell extracts^29^ using a combination of heat treatment, anion exchange chromatography on a HiTrap Q HP column from GE Healthcare, and gel filtration on a Superdex 200 10/300GL (GE Healthcare).

### Enzyme Assay

Kinetic analyses of the new variant aldolases (Variants 3 and 4) were carried out using a modification of the thiobarbituric acid assay.^21^ Assays were carried out at 70 °C in 50 mM sodium phosphate buffer, pH 6.0, using various concentrations of pyruvate and d- and l-glyceraldehyde. Aldol reactions were started by the addition of enzyme and stopped at known time intervals by the addition of 12% (w/v) trichloroacetic acid, with the presence of any 3-deoxy-hexulosonic acid products then detected using the thiobarbituric acid assay.

### Determination of the Diastereoselectivities of Aldolase-Catalyzed Reactions

Biotransformation reactions between 600 mM pyruvate and 100 mM either d-glyceraldehyde or l-glyceraldehyde were carried out at 50 °C in water using the four variant aldolases (10 μg). The diastereomeric ratios of the aldol products produced in these aldol reactions were then determined by high-pressure liquid chromatography (HPLC) analysis using an Agilent 1200 machine fitted with a BioRad Aminex HPX-87H column (300 mm × 7.8 mm) running at 0.4 mL/min in 8 mM H_2_SO_4_ at 60 °C, with peak detection carried out using a refractive index detector.

### Biotransformations

The variant aldolases 1, 2, 3, and 4 were used to prepare d-KDGlc, d-KDGal, l-KDGlc, and l-KDGal, respectively, using our previously published methods.^23,29^ Enantiopure d- or l-glyceraldehyde (150 mg, 1.67 mmol) and sodium pyruvate (1.00 g, 9.00 mmol) were dissolved in 100 mL of water containing 10 mg of the appropriate aldolase variant. The reaction was heated to 50 °C in a shaking incubator. When consumption of glyceraldehyde was sufficient (monitored by HPLC, see the conditions above), the reactions were lyophilized to produce 0.7–1.0 g of crude product. Portions of the crude product (100 mg) were then purified by semipreparative HPLC using a Bio-Rad Aminex HPX-87H column to remove excess pyruvate, with yields of up to 20 mg (per portion) of each purified 3-deoxy-hexulosonic acid obtained in >95:5 dr. Alternatively, purification and separation of diastereomers could be carried out by anion exchange chromatography (0–0.6 M aqueous formic acid, Dowex 1X8-formate resin). See the Supporting Information for representative characterization data.

### Crystallization and X-ray Data Collection and Processing

Variants were crystallized by the hanging-drop vapor diffusion method at 18 °C. Substrate complexes were obtained by soaking crystals in 30 mM solutions of the 3-deoxy-hexulosonic acid. Variant 2 was cocrystallized with pyruvate by adding 100 mM pyruvate to the crystallization conditions. Glycerol (15–20%, v/v) was added to the crystals as a cryoprotectant prior to data collection. X-ray diffraction data were collected at the Diamond Light Source (experiments mx1226-10, mx1226-11, and mx1226-23) or in-house using Cu Kα radiation from a Rigaku MicroMax 007HF generator using a Saturn 944+ detector. Data were processed initially with DIALS, but if this was unsuccessful, they were then processed with MOSFLM, or if necessary with HKL2000 as a final choice, to obtain data with the lowest *R*_merge_ and the highest completeness. The wild-type aldolase structure (1W37) was used to solve the variant structures using BALBES,^30^ with the models subsequently refined using Refmac^31^ or Phenix.^32^

## Results and Discussion

### Characterization of SsKDG-Aldolase and Its Variants

The approach used to construct Variant 1 (Y132V/T157C) and Variant 2 (T157V/D181Q/A198L) aldolases that exhibit selectivity for formation of d-KDGlc and d-KDGal, respectively, has been described previously.^29^ Briefly, X-ray crystallographic structures of the wild-type aldolase bound to d-KDGlc and d-KDGal were used to identify specific active site amino acids responsible for producing stabilizing interactions to each of the aldol diasteroisomers (Figure 2). These residues were then targeted for mutation with the aim of disrupting stabilizing active site binding interactions that were responsible for binding d-KDGlc (or d-KDGal). This approach resulted in rapid identification of a Variant 1 mutant that favored formation of d-KDGlc in 93:7 dr and a Variant 2 mutant that produced d-KGal in 88:12 *dr*. A similar strategy (outlined in detail below) has been used in this study to create Variant 3 (T157V/A198L) and Variant 4 (Y103F/Y130F/A198F) mutants that exhibit complementary diastereoselectivity profiles for formation of non-natural l-KDGlc and l-KDGal, respectively. The kinetic parameters of the wild-type and variant aldolases used in this study were determined using a modified thiobarbituric acid assay, as described in the Methods and Materials section. Each variant was assayed with each enantiomer of glyceraldehyde, with the dr of their aldol products then determined by HPLC analysis (HPLC traces are shown in Figure S1).

The enzyme kinetic and dr data obtained for the variants are summarized in Table 1. It should be noted that the *K*_M_ values are operational values; that is, they are the substrate concentrations giving half *V*_max_ in the presence of near-saturating (≥90%) concentrations of the second substrate. The precise constants comprising these *K*_M_ values will depend on the mechanism of the catalyzed reaction (random rapid equilibrium or compulsory order).

**Table 1:** Kinetic Parameters of SsKDG-Aldolase and Its Variants^a^

	*K*_M_ (mM)	*V*_max_ (μmol/min/mg)	dr of aldol products KDGlc:KDGal
wild-type			
pyruvate	1.1 (±0.1)	8.3 (±0.1)	55:45
d-glyceraldehyde	5.3 (±0.3)	8.9 (±0.3)	
pyruvate	0.5 (±0.1)	5.4 (±0.1)	53:47
l-glyceraldehyde	3.8 (±0.1)	5.6 (±0.04)	
Variant 1			
pyruvate	4.1	0.13	93:7
d-glyceraldehyde	8.0	0.11	
Variant 2			
pyruvate	39.6	0.12	12:88
d-glyceraldehyde	9.1	0.13	
Variant 3			
pyruvate	6.0 (±0.2)	5.7 (±0.1)	92:8
l-glyceraldehyde	37.6 (±0.2)	6.1 (±0.1)	
pyruvate	2.9 (±0.1)	1.7 (±0.02)	28:72
d-glyceraldehyde	19.5 (±0.2)	1.8(±0.01)	
Variant 4			
pyruvate	1.1 (±0.15)	0.06 (±0.01)	24:76
l-glyceraldehyde	6.3 (±0.18)	0.06 (±0.01)	
pyruvate	0.3 (±0.04)	0.09 (±0.01)	75:25
d-glyceraldehyde	6.9 (±0.2)	0.11 (±0.01)	

a Data for the wild-type enzyme and for Variants 1 and 2 are taken from Royer et al.^29^ and are included for comparison with the data generated in the current paper for Variants 3 and 4. The errors given are for two repeats of each experiment (*n* = 3).

The synthetic utility of the SsKDG-aldolase variants as biocatalysts has been demonstrated by using them to produce their respective aldol products for use as docking substrates to carry out aldolase-ligand crystallization studies.

### Crystallization of SsKDG-Aldolase Variants

Crystals of the various SsKDG-aldolase variants were obtained using 11–15% (v/v) PEG4K, 4–12% (v/v) isopropanol, and 0.1 M HEPES, pH 5.9–6.8. Data were collected for each of the four uncomplexed variants, with subsequent data sets then collected for each variant following their soaking in a 30 mM concentration of their preferred 3-deoxy-hexulosonic acid ligand (major aldol product), with soaking times ranging from 1 min to 24 h. Unfortunately, crystals of a complex of Variant 2 docked with d-KDGal could not be obtained, even after soaking was carried out for 24 h, although its cocrystallization with pyruvate was successful. Data collection and processing statistics for Variants 1–4 are presented in Table S1.

### Structural Analysis of the Wild-Type SsKDG-Aldolase Bound to d-KDGlc and d-KDGal

The previously reported X-ray crystal structure of the wild-type SsKDG aldolase–d**-**KDGlc complex (PDB code 1W3N) revealed that the 4-OH group of d**-**KDGlc forms hydrogen bonds with the phenol and hydroxyl groups of the Y130 and T157 residues, with its 6-OH group interacting with the phenol group of Y132 (Figure 2A). The crystal structure of the wild-type enzyme complexed with d**-**KDGal (PDB code 1W3T) revealed that d**-**KDGal makes strong hydrogen bonds between the 4-OH and the T157 hydroxyl group, with its 5-OH group interacting with the Y132 phenol group and its 6-OH hydroxyl group interacting with the T44 hydroxyl group (Figure 2B). With this structural information in hand, it was proposed that the d**-**KDGlc selectivity observed for Variant 1 (Y132V/T157C) was due to elimination of stabilizing contributions from Y132 and T157 in the aldol transition state that favors d**-**KDGal formation in the wild-type enzyme.

Similar arguments were used to explain the improved d-KDGal selectivity of Variant 2 (T157V/D181Q/A198L), which was suggested to be due to disruption of stabilizing hydrogen bond interactions as well as the introduction of a hydrophobic region in the binding site that disfavors the transition state leading to d-KDGlc formation in the wild type. Consequently, we decided to seek further structural evidence for these mechanistic explanations by acquiring the crystal structures of Variant 1 complexed to d-KDGlc and Variant 2 complexed to d-KDGal, whose structures would then be compared to those of the wild-type aldolase bound to d-KDGlc and d-KDGal.

### Structural Analysis of the d-KDGlc Selective Variant 1 (Y132V/T157C)

As previously reported,^29^ the effect of introducing the Y132V and T157C mutations into Variant 1 is to increase selectivity for d-KDGlc production from 55:45 to 93:7 dr (Table 1). The overall structure of this variant was found to be very similar to the wild-type enzyme (0.3 Å RMSD over all 293 Cα positions for Variant 1 and 0.4 Å for the Variant 1–d-KDGlc complex obtained at a lower resolution). This indicates that the mutations in Variant 1 do not cause major structural disruption, with the only significant movements (more than 3× RMSD) occurring at the mutation sites. Comparison of the unsoaked structure (2.0 Å resolution) of Variant 1 with the wild-type aldolase revealed that the only major side-chain movement was of Y130, which moves 1.5 Å away from K155. This is potentially significant because the T157C mutation of Variant 1 means that Y130 is the only remaining amino acid capable of forming stabilizing interactions with the 4-OH groups of both d-KDGlc and d-KDGal. An unexpected consequence of the mutations in Variant 1 was the formation of a strong interaction between the Nζ amino group of K155 (the Schiff-base forming lysine residue) to the introduced Sγ thiol group of C157 (Figure S2). This potentially strong interaction needs to be disrupted for catalysis to occur, which, combined with displacement of Y130 (that also plays a role in the catalytic mechanism),^25,33^ potentially explains the lower *V*_max_ value of this variant (Table 1). The presence of this K155–C157 interaction may also explain the difficulties experienced in obtaining a complexed structure, with a 24-hour soak required to obtain a low-resolution complex (3.15 Å) of Variant 1 with d-KDGlc, with strong electron density for bound d-KDGlc only seen in the active site of one of the two aldolase molecules present in the crystal unit cell.

**Figure 4:**
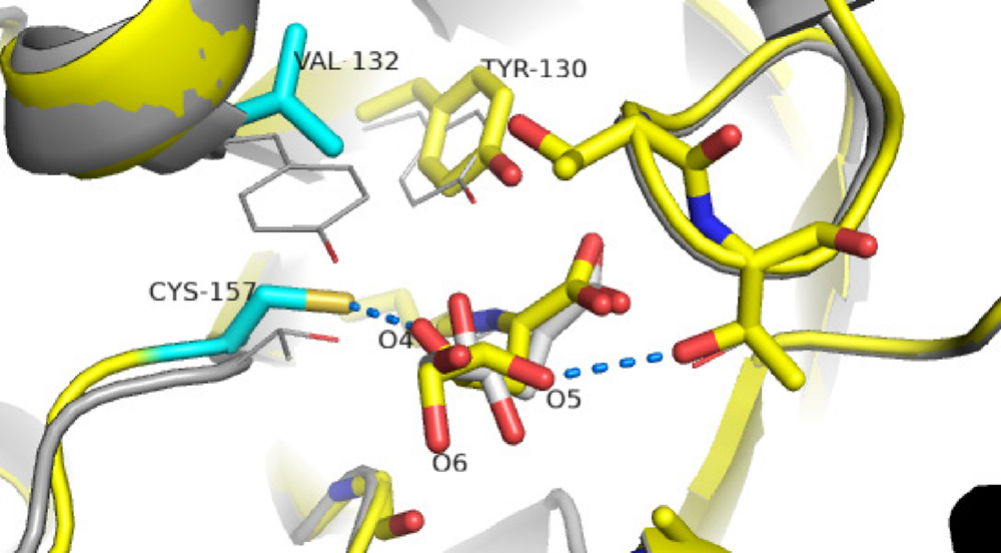
Active site of Variant 1 in complex with d-KDGlc superimposed onto the corresponding d-KDGlc complex of the wild-type enzyme. Variant 1 amino acids are shown as yellow sticks, with mutations shown in cyan. Conformations of d-KDGlc in wild-type (gray) and Variant 1 (yellow) complexes are shown, together with stabilizing hydrogen bond interactions (blue dotted lines) between O4 and O5 of d-KDGlc and residues in the active site.

As seen in the other variants, examination of the structure of Variant 1 complexed with d-KDGlc revealed that interactions between the active site residues and the acid region of d-KDGlc were essentially unchanged when compared to the wild-type structures (Figures 4 and S3, upper part of the bound substrate). A relatively short distance (2.35 Å) between the 4-OH of bound d-KDGlc and the Sγ thiol of C157 was observed in this variant, with the corresponding distance in a KDGal complex predicted to be even shorter (compared to the short distance between O4 of d-KDGal and T157 in the wild-type enzyme). This agrees with our previous suggestion^29^ that the sterically demanding Cys residue in Variant 1 should disfavor d-KDGal formation. Further evidence that steric interactions with C157 may be responsible for the change in specificity is that the corresponding T157S variant has specificity almost unchanged from WT, while the T157C single mutant produces 75% dr favoring d-KDGlc.^29^ The movement of Y130 away from K155 removes any interactions with the 4-OH group of d-KDGlc in Variant 1, with Y130 now only interacting with the O1 carbonyl of d-KDGlc, which means that it is unlikely to affect the diastereoselectivity of the Variant 1 aldol reaction.

Potential stabilizing interactions between the 5-OH and 6-OH groups of KDGlc and the phenol group of Y132 in the wild-type enzyme are removed through introduction of the Y132V mutation into Variant 1, which results in the C4–C5 bond of d-KDGlc rotating to enable a new hydrogen bond between the 5-OH group and the T44 hydroxyl residue. A hydrogen bond between the 6-OH and T44 hydroxyl group already exists in the wild-type enzyme–d-KDGal complex, so this interaction does not contribute additional stabilization to the transition state leading to d-KDGal formation. Therefore, destabilizing steric interactions between the C157 residue and the d-KDGal 4-OH hydroxyl group and removal of stabilizing hydrogen bonding interactions between the Y132 phenol group and the d-KDGal 4-OH and 5-OH groups, coupled with a new stabilizing interaction between the d-KDGlc 5-OH and T44 hydroxyl group, result in Variant 1 favoring the selective formation of d-KDGlc in 93:7 dr.

### Structural Analysis of the d-KDGal Selective Variant 2 (T157V/D181Q/A198L)

As previously reported,^29^ the effect of introducing T157V, D181Q, and A198L mutations into Variant 2 results in a mutant that catalyzes selective formation of d-KDGal in 88:12 dr, which is a considerable increase over the 45% selectivity for d-KDGal produced by the wild-type aldolase. The kinetic parameters of Variant 2 are similar to those of Variant 1, (i.e., a similarly reduced *k*_cat_) with the exception of its *K*_M_ for pyruvate, which was 10-fold greater than Variant 1 and 40-fold greater than that for the wild-type enzyme (Table 1).

X-ray crystallographic data for both the unsoaked (1.57 Å) and the pyruvate-complex structures (2.17 Å) were collected at a higher resolution than was achieved for Variant 1; however, these were in a space group *P*2_1_ not seen in the other variants. Density for the Schiff base lysine (K155) could not be observed, even when the variant was cocrystallized with pyruvate, and no complexes of Variant 2 with d-KGal were obtained from multiple attempts.

**Figure 5:**
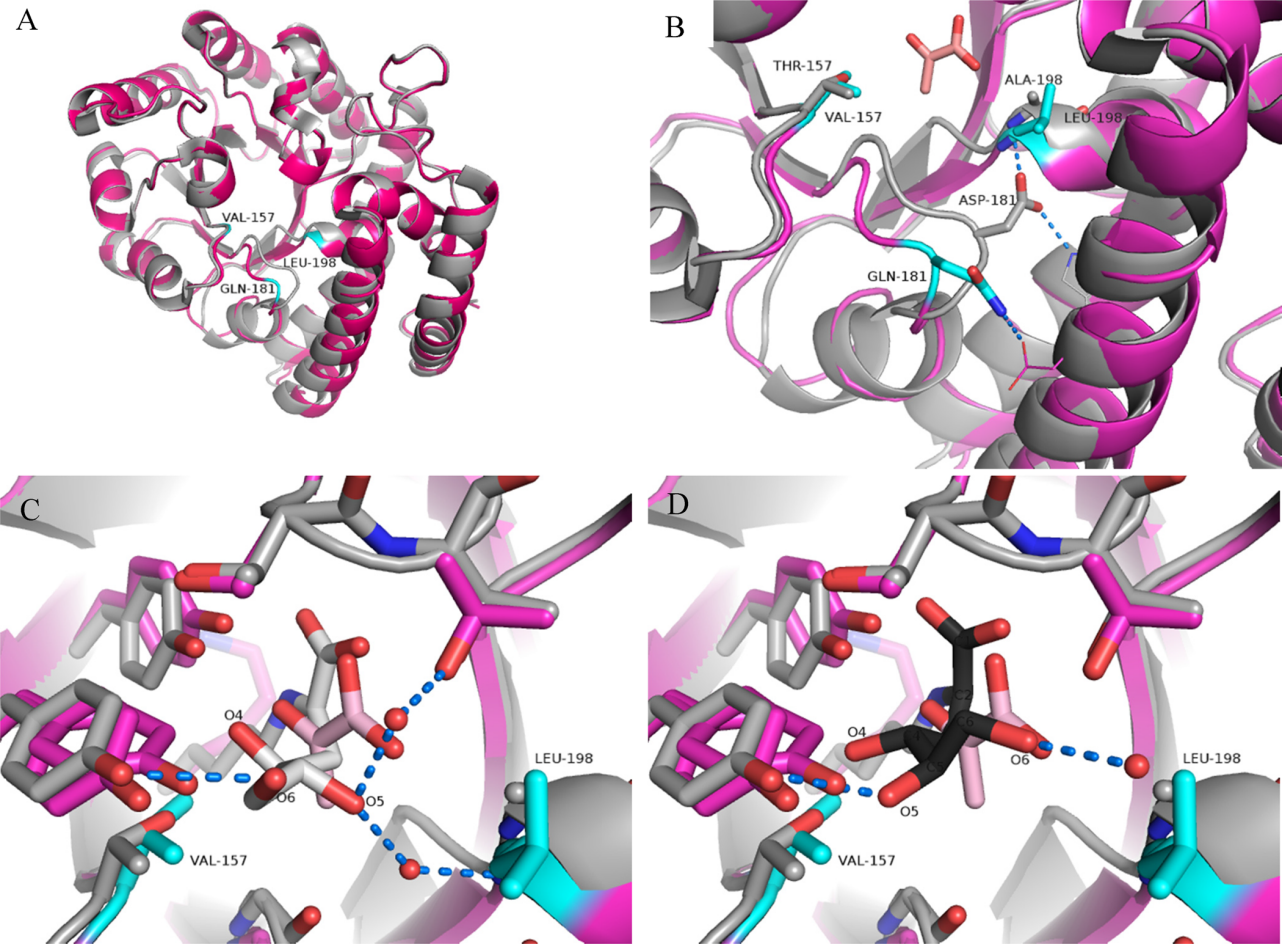
(A) Superimposition of the Variant 2 (magenta) and wild-type (gray, PDB code: 1W37) SsKDG-aldolase structures with mutation sites labeled and colored in cyan. Major movements occur in the lower middle portion of figure (A), which are magnified in figure (B). Pyruvate, bound in the variant structure, is shown in pink. Hydrogen bonds are shown by blue dotted lines. The active site of Variant 2 and the wild-type enzyme structure (gray) shown in complex with (C) d-KDGlc (white, PDB code: 1W3N) or (D) d-KDGal (black, PDB code: 1W3T), both of which show displacement of pyruvate within the Variant 2 active site. Water molecules involved in hydrogen bonds in the wild-type enzyme are shown as red spheres. Hydrogen bonds in figures (C and D) for the 5-OH and 6-OH groups of KDGlc and KDGal are those present in the corresponding wild-type SsKDG-aldolase structures.

The four molecules of the tetramer within the Variant 2 structure are very similar, having an average RMSD within the uncomplexed structure of only 0.2 Å (and between this structure and the pyruvate complex); however, the 0.7 Å difference when compared to the wild-type aldolase was larger than those for the other variants. Looking at the superimposed structures (Figure 5A), most of the wild-type and Variant 2 residues align to a much greater degree than might be expected from these RMSD values. However, significant displacement of the main chain backbone between residues 180 and 191 is seen, which is presumably caused by the D181Q mutation (Figure 5B). We had previously suggested^29^ that inclusion of a longer glutamine residue for D181 in the loop underneath L198 (see the orientation shown in Figure 5B) would restrict the conformational freedom of this leucine residue, thus making this region of the active site more hydrophobic. This structural change was predicted to promote preferential binding of d-KDGal over d-KDGlc by Variant 2 because it would disrupt water-mediated hydrogen bonds present in the active site that we predicted would be more important for producing d-KDGlc over d-KDGal in the wild-type enzyme. The crystal structure of Variant 2 revealed that the primary amide group of the Q181 residue no longer makes interactions with the amide group of L198 (as seen in the wild-type enzyme), with Q181 now pointing in the opposite direction to form a hydrogen bond with the acid group of D230. This removes any conformational restraints on the L198 loop region of the active site (Figure 5B), which can relax away from the catalytic center, resulting in the floor of the active site being lowered below the pyruvate binding site. This relatively large conformational change may explain this variant’s significantly greater *K*_M_ for pyruvate.

Comparison of the structure of Variant 2 (cocrystallized with pyruvate) with the wild-type enzyme complexed with d-KDGlc and d-KDGal (Figure 2) reveals that pyruvate is displaced from its active position, making no hydrogen bonds with T43, (Figures 5C and S4) while electron density for the Schiff base K155 is not visible in the active site. Nevertheless, our original hypothesis that was proposed to explain the d-KDGal selectivity of Variant 2 appears to still be valid. Replacement of T157 by a hydrophobic valine group will remove stabilizing hydrogen bond interactions to the 4-OH groups of both d-KDGlc and d-KDGal. However, introduction of the Leu198 residue will create a new hydrophobic region in the active site, which is likely to displace proximal active site water molecules. These bridging water molecules provide greater stabilizing hydrogen bond interactions with both the 5-OH and 6-OH groups of bound d-KDGlc than with the single 6-OH group of bound d-KDGal (whose 5-OH is also stabilized by interactions with T192) in wild-type enzyme complexes. Therefore, their absence in Variant 2 is likely to disfavor d-KDGlc formation, thus resulting in preferential d-KDGal in 88:12 dr (Figure 5).

### Structural Analysis of the l-KDGlc Selective Variant 3 (T157V/A198L)

Identification of an SsKDG-aldolase variant that could selectively transform non-natural l-glyceraldehyde into l-KDGlc began by screening the selectivity profile of the library of T157 saturation mutants generated previously to identify Variants 1 and 2.^29^ This identified that T157V (used in Variant 2 to produce d-KDGal selectivity) was a suitable starting point for generating l-KDGlc selectivity, with inclusion of a second substitution (A198L) to create a hydrophobic pocket to disrupt the proximal hydrogen bonding water network (as used in the design of Variant 2) affording Variant 3 that produced l-KDGlc in 92:8 dr. The kinetic parameters of this variant were then determined (Table 1), with its *V*_max_ found to be very similar to that of the wild-type aldolase; however, the *K*_M_ values of Variant 3 for both aldol substrates were found to be increased. Crystallization of Variant 3 with l-KDGlc was carried out under standard conditions; however, its higher activity than those of Variants 1 and 2 meant that effective trapping of a ligand complex required shorter soaking times. For instance, a 4-min soak of Variant 3 with l-KDGlc resulted in a covalent Schiff-base lysine complex of pyruvate (formed from retro-aldol cleavage of l-KDGlc; data not shown). However, data acquired after a 2 min soak revealed greater continuous electron density within the active site, which enabled us to determine the structure of the l-KDGlc complex (Figure S5).

**Figure 6:**
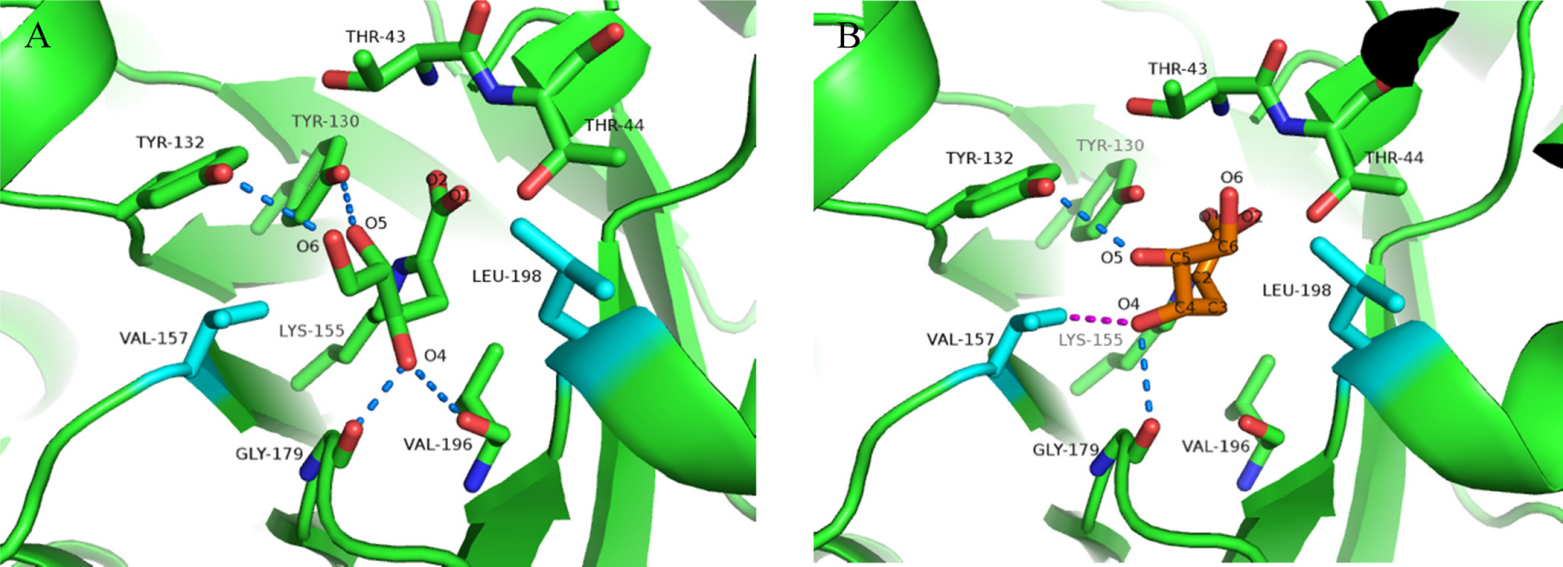
Active site structure of SsKDG-aldolase Variant 3 shown in green, with mutated residues shown in cyan. (A) l-KDGlc is shown having formed a Schiff-base link to Lys155, with key hydrogen bonds shown by blue dotted lines. (B) Superimposition of the conformation of l-KDGal (orange) from the Variant 4–l-KDGal complex (see Figure 7) onto the Variant 3 active site reveals that the 4-OH group of l-KDGal is likely to make unfavorable hydrophobic interactions with Val157 (magenta dotted line).

The structure of Variant 3 is essentially unchanged from the wild-type enzyme (RMSD of both soaked and unsoaked structures compared to the wild-type structure 1W37 is 0.3 Å, as are the RMSD between molecules within these structures). Unfortunately, structures of the wild-type SsKDG-aldolase complexed with l-KDGlc or l-KDGal were not available for comparison with the Variant 3–l-KDGlc structure. However, comparison with the wild-type enzyme complexed with d-KDGal (which has the same (*R*)-configuration as l-KDGlc at O4) (Figure 2) revealed that the 4-OH of l-KDGlc in Variant 3 had rotated away from V157 to make a new hydrogen bond interaction with the main chain peptide bonds of G179 and V196 at the bottom of the active-site cleft (Figure 6). This results in the 5-OH and 6-OH groups of l-KDGlc now making hydrogen bonds with the phenol groups of Y130 and Y132, respectively. This conformational realignment means that l-KDGlc presents a nonpolar face to the introduced V157 and L198 residues, which both combine to form a hydrophobic sandwich on either side of the active site.

Comparison of the structures of the Variant 3–l-KDGlc complex to the Variant 4–l-KDGal complex (vide infra) revealed that the 4-OH group of l-KDGal would be predicted to make unfavorable hydrophobic interactions with Val157 in Variant 3 (Figure 6B).

Rotation of the 4-OH group of l-KDGal to relieve this unfavorable interaction (to occupy a position similar to that observed for the 4-OH group of docked l-KDGlc in Variant 3) would result in its 5-OH group being brought into close proximity to V157, which is equally unfavorable. Therefore, the ability of l-KDGlc to present a hydrophobic face to the introduced hydrophobic amino acids (V157 and L198 ), while still maintaining multiple stabilizing hydrogen bonding interactions between its 5-OH and 6-OH hydroxyl groups, most likely explains the selectivity of Variant 3 for l-KDGlc formation in 92:8 dr. The presence of the two hydrophobic amino acids (V157 and L198) is likely to also restrict the overall conformational mobility of the aldehyde substrates within the active site of Variant 3, which may explain its higher *K*_M_ for l-glyceraldehyde.

### Structural Analysis of the l-KDGal Selective Variant 4 (Y103F/Y130F/A198F)

Producing a variant with a significant preference for l-KDGal over l-KDGlc proved challenging, with most structure-guided variants explored proving nonselective for formation of l-KDGal or displaying precipitous losses in catalytic activity. Saturation mutagenesis at T157 did not reveal a lead variant with l-KDGal selectivity. The other residue that interacts with O4 is Y130; however, this residue is responsible for donating a proton to O4 in the wild-type mechanism.^25,33^ Nonetheless, it was decided to explore the activity of a Y103F variant, in the hope that the Y99 side chain could mimic the role of the Y130 OH residue of the wild-type enzyme. Further specificity was achieved through replacement of the A198 residue with a hydrophobic phenylalanine residue to disrupt the previously discussed hydrogen bonding water network that favored d-KDGlc formation. This triple mutant Variant 4 (Y103F/Y130F/A198F) was shown to selectively transform l-glyceraldehyde into l-KDGal in 76:24 dr (cf 47:53 dr in the wild-type aldolase).

**Figure 7:**
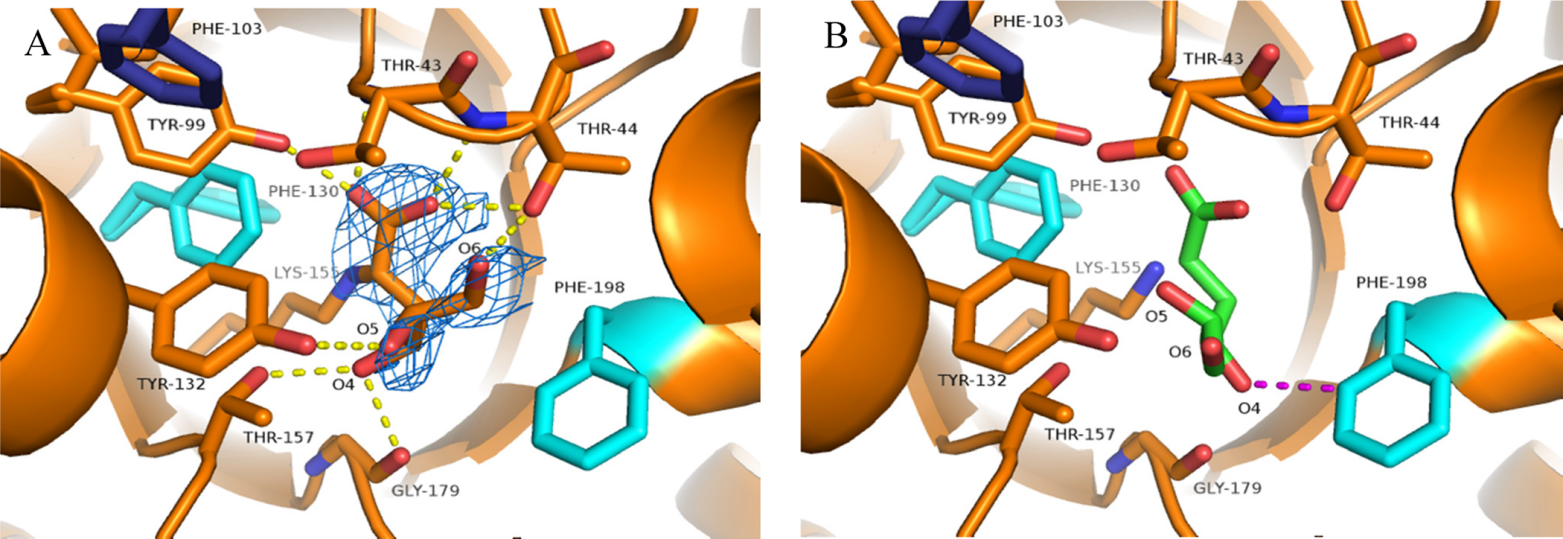
(A) Structure of SsKDG-aldolase Variant 4 with ligand-interacting active-site residues shown in orange. Mutated amino acids in the same monomer are shown in cyan, with mutated amino acids from a different aldolase monomer shown in dark blue. 2FoFc density (contoured at 1σ) shown around the bound l-KDGal residue. Stabilizing hydrogen bond interactions are indicated by yellow dotted lines. (B) Variant 4 structure with l-KDGlc conformation present in the Variant 3–l-KDGlc complex superimposed. Close proximity of the destabilizing 4-OH group of the bound l-KDGlc to Phe198 is shown by the magenta dotted line.

**Figure 8:**
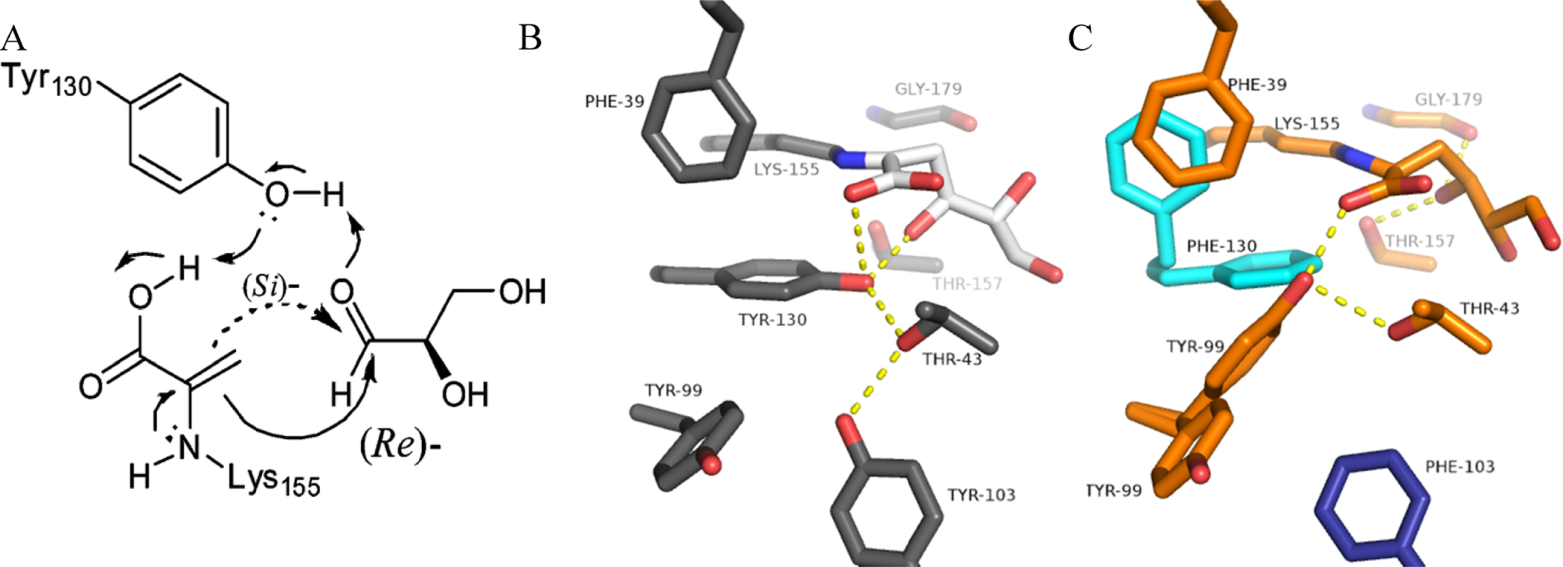
(A) Y130 residue of the wild-type aldolase is intimately involved in facilitating reaction of the K155-derived enamine derivative of pyruvate at the *Re*- or *Si*-face of d-glyceraldehyde to afford d-KDGlc or d-KDGal.^25,33^ (B) Positions of amino acids around Y130 in the wild-type SsKDG-aldolase structure shown in gray, with bound d-KDGlc shown in white. Hydrogen bonds made by the phenol group of Y130 are shown as yellow dotted lines. (C) Positions of equivalent amino acids in Variant 4 shown in orange, with mutated residues in the same monomer shown in cyan and residues from a neighboring aldolase monomer shown in dark blue. Hydrogen bonds made by the phenol group of the replacement Y99 and the 4-OH group of l-KDGal are shown as dotted yellow lines.

The crystal structure of the wild-type enzyme–d-KDGal complex reveals that the Y130 residue (together with T157) interacts with the 4-OH group in the wild-type SsKDG–aldolase complexes (Figure 2); however, Y130 is also intimately involved in the catalytic mechanism of the aldolase (see Figure 8). Consequently, removal of the stabilizing Y130 phenol group significantly affected the catalytic activity of Variant 4, resulting in a marked reduction in its *k*_cat_ value (see Table 1). However, Variant 4 still retained sufficient catalytic activity to be useful as a biocatalyst for the synthesis of synthetically useful amounts of l-KDGal. The main chain structure of the active site of Variant 4 remains unchanged on binding to l-KDGal, as shown by an RMSD of only 0.3 Å between Variant 4 and the wild-type structure and between the structures of soaked and unsoaked Variant 4. Comparison of the structure of Variant 4 bound to l-KDGal to the wild-type structure bound to d-KDGlc (Figure 2A, same (*R*)-4-OH configuration) revealed that the 4-OH group of l-KDGal in Variant 4 rotates away from F130 to make a new hydrogen bond with the backbone amide of G179 while still maintaining an interaction with the T157 hydroxyl group (Figure 7A). This enables the 5-OH and 6-OH groups of Variant 4-bound l-KDGal to make hydrogen bonds directly to both the phenol group of Y132 and the hydroxyl group of T44, which results in l-KDGal presenting a hydrophobic face toward the aryl ring of the introduced F198 residue.

Superimposition of the structure of l-KDGlc–Variant 3 onto l-KDGal–Variant 4 reveals that the 4-OH hydroxyl group of l-KDGlc likely occupies a position close to F198 when docked into Variant 4 (Figure 7B), and so its binding energy would be predicted to be significantly higher than the binding energy required to accommodate l-KDGal. Rotation of the C3–C4 bond of l-KDGlc to allow its 4-OH group to adopt a similar conformation to the 4-OH group of bound l-KDGal (i.e., to allow hydrogen bonding interactions with T157 and G179) would result in the l-KDGlc 5-OH group being brought into unfavorably close proximity to the hydrophobic F198 residue.

Therefore, removal of the 4-OH-Y130 hydrogen bond from Variant 4 that preferentially favors KDGlc formation in the wild-type enzyme, combined with the presence of a new hydrophobic pocket formed by the A198F mutation, results in Variant 4 promoting selective formation of l-KDGal in 76:24 dr. While the proposed structural interpretation can be used to explain the observed shift in l-KDGal selectivity from 55:45 in the wild-type to 24:76 in Variant 4, it is recognized that this difference in diastereoselectivity corresponds to a relatively small difference in activation free energy (∼0.8 kcal/mol, vide infra), and so these differences in diastereocontrol may equally be caused by other secondary effects.

As previously reported, the mechanism of the wild-type SsKDG-aldolase-catalyzed reaction involves direct interactions between the Y130 phenol residue and the pyruvate and glyceraldehyde substrates (see Figure 8A).^25,33^ This Y130 residue also forms a direct hydrogen bond with the T43 residue, which interacts with the Y103 residue of another aldolase monomer, thereby establishing a stabilizing hydrogen bonding network of tyrosine/threonine residues that has been observed in other aldolases. These amino acids are well defined in the wild-type aldolase structure, with each residue exhibiting relatively low mobility to produce an effective hydrogen bonding network that facilitates catalysis (Figure 8A). Two of these tyrosine residues (103 and 130) have been mutated to phenylalanine residues in Variant 4, with the residual F130 residue observed to adopt two conformations: a conformation similar to the one seen in the wild-type enzyme and a new conformation stabilized through a π-stacking interaction with F39 (Figure 8B). Movement of the F130 side chain out of the active site, together with replacement of Y103 by a hydrophobic phenylalanine residue, results in relaxation of the active site, which allows the Y99 residue to adopt a new conformation that places its phenolic group in a similar position to that previously occupied by the Y130 residue of the wild-type enzyme. Therefore, this conformational rearrangement in the Variant 4 active site appears to be sufficient to enable Y99 to participate in the aldol reaction of pyruvate and l-Glyceraldehyde to selectively afford l-KDGal, albeit at a significantly reduced rate when compared to the corresponding aldol reaction of the wild-type enzyme. Although Y99 can interact directly with T43 and the C1 carbonyl of l-KDGal, it is likely to be too remote from the 4-OH group to provide a stabilizing interaction. Consequently, the 4-OH group of l-KDGal rotates to make interactions with the hydroxyl group of T157 and the backbone amide group of G179 (Figure 8C), with this nonoptimal orientation also likely to contribute to the considerably lower reaction rate of Variant 4.

### Factors Affecting the Diastereoselectivities of the Aldol Reactions of Wild-Type KDG-Aldolase and Its Variants

The preceding sections describe how subtle changes in active site interactions of Schiff-base-linked Lys155-bound KDGlc and KDGal aldol products of the wild-type aldolase and its mutant variants have been used to rationalize the diastereoselectivities of the respective aldol reactions responsible for their formation. The drs of the aldol products produced by the variants could potentially be caused by differences in aldehyde substrate-binding geometries prior to carbon–carbon bond formation and/or differences in the free energy barriers of the transition states leading to formation of the KDGlc and KDGal diastereomers.^26^ Consequently, it was decided to explore the binding preferences of the wild-type and variant aldolases using molecular dynamic simulations of each variant containing a Lys155-pyruvate Schiff base and a bound glyceraldehyde substrate. Simulations were performed using d- and l-glyceraldehydes as substrates for the wild-type enzyme, with d-glyceraldehyde used for Variants 1 and 2, and l-glyceraldehyde used for Variants 3 and 4 (see the Supporting Information for details). None of these simulations produced an excess of pre-*S* or pre-*R* glyceraldehyde-bound conformations that could explain the experimentally determined dr levels of either the wild-type enzyme or its variants (Figures S6 and S7). Therefore, it follows that the observed differences in the diastereoselectivities of each variant’s aldol reaction must arise from differences in the transition-state energies of the *anti*- and *syn*-aldol reactions that lead to their respective KDGlc and KDGal products [as rationalized by the X-ray crystallographic analyses (vide supra)]. This conclusion is not unexpected, as the structures of the docked variant-aldol products are likely to resemble the transition states of the aldol reactions that lead to their formation quite closely, with the C3–C4 bond distance in their transition states expected to be ∼2.1 Å.^27^ The differences in the energy barriers of the transition states that produce the observed dr levels of each variant are relatively small, corresponding to a difference of 0.8 to 1.8 kcal/mol depending on the dr value of the variant (determined from Δ*G* = −*RT*ln(*Z*), where *Z* is the diastereomeric ratio at 70 °C).

Examination of the structures of the wild-type aldolase bound to d-KDGlc and d-KDGal reveals that their 4-OH groups both make hydrogen bonds to the OH groups of Y130 and T157. Assuming that similar interactions occur in the transition states leading to their formation, similar two-point hydrogen bond interactions with the electron lone pairs of the carbonyl group of d-glyceraldehyde will activate it toward nucleophilic attack by the K155-bound pyruvate nucleophile. The nonselective aldol reaction of the wild-type enzyme requires that the incoming K155-bound pyruvate nucleophile has approximately equal access to both the *Re*- and *Si*-faces of the bound aldehyde group (as indicated by the MD simulations) and approximately equal energy barriers for reaction. This is achieved by binding d-glyceraldehyde in different pre-*R* and pre-*S* conformations that are stabilized by different hydrogen bonding interactions between the 2-OH and 3-OH residues of d-glyceraldehyde (5-OH and 6-OH in KDGlc and KDGal) and different amino acid residues and/or bound water molecules within the active site. Therefore, the promiscuity of the wild-type enzyme is due to its ability to access different hydrogen bond networks that stabilize transition states with the pyruvate nucleophile attacking either the *Si-* or *Re-*face of the aldehyde group that generate d-KDGlc and d-KDGal, respectively. For the wild-type enzyme, the energy differences between these pre-*R* and pre-*S* derived transition states leading to d-KDGlc and d-KDGal are negligible, which leads to formation of mixtures of aldol products in low 55:45 dr favoring d-KDGlc.

Examination of the structure of d-KDGlc-bound Variant 1 indicates that the aldehyde group of d-glyceraldehyde is predicted to be activated through a new hydrogen bond to C157, with its 5-OH and 6-OH residues then making different interactions with active site hydrogen bond acceptors that favor d-KDGlc formation. No ligand-bound structures could be obtained for Variant 2; however, the structure of Variant 3 bound to l-KDGlc indicates that the aldehyde group of l-glyceraldehyde in the transition state leading to l-KDGlc (major aldol product) should be activated toward nucleophilic attack through hydrogen bonding to the amide bonds of G179 and V196. Alternatively, the structure of Variant 4 bound to l-KDGal reveals that the aldehyde group of l-glyceraldehyde in the transition state leading to l-KDGal (major aldol product) should be activated by hydrogen bonding to the hydroxyl group of T157 and the amide bond of G179. Therefore, Variants 3 and 4 bind the carbonyl group of l-glyceraldehyde in different regions of their active sites when compared to the bound carbonyl group of d-glyceraldehyde in the active sites of the wild-type enzyme and Variant 1.

The ligand binding structures shown in Figure 2 reveal that the wild-type KDGlc aldolase can contribute multiple hydrogen bond acceptor residues and bridging water molecules to enable its d- and l-glyceraldehyde substrates to be bound effectively. Therefore, different networks of hydrogen bond acceptors can be used to bind either glyceraldehyde in different pre-*R* and pre-*S* conformations that can each react with the K155-bound pyruvate nucleophile through transition states that lead to KDGlc and KDGal products. In the wild-type enzyme, the difference in the energies of the pre-*R* and pre-*S* derived transition states must be small, which means that the KDGlc and KDGal diastereomers are produced in similar amounts. Selective mutation of active-site residues to generate Variants 1–4 results in mutants whose active sites make different H-bonding interactions with the carbonyl and hydroxyl groups of their bound glyceraldehyde substrates. The different hydrogen bonding networks of each variant then serve to perturb the relative energy differences of the transition states leading to their l/d-KDGlc and l/d-KDGal products, thus allowing selective production of a different major aldol diastereomer in each case.

We believe that our strategy of selectively introducing or deleting active site residues that make selected hydrogen bond interactions with the polar substituents of an aldehyde substrate may prove to be a generally useful approach for improving the specificity and stereoselectivity profiles of other types of aldolase. Although we have shown that this approach leads to significant decreases in activity, the generally high activities of many aldolases indicates that variants with lower activities but good levels of stereocontrol can be employed for the synthesis of useful quantities of enantiopure aldol products. While this study has been directed toward evolving the stereoselectivity of the KDG aldolase toward polar hydroxy-aldehyde substrates, we anticipate that a similar active site editing strategy may be useful for conferring aldol reactivity and stereoselectivity toward more hydrophobic aldehyde substrates.

## Conclusions

Toscano et al.^15^ have remarked that “enzyme active sites provide highly optimized microenvironments for the catalysis of biologically useful chemical transformations. Consequently, changes at these centers can have large effects on enzyme activity.” In their review, they describe how minimal modifications to enzyme active sites can expand their catalytic abilities to give both enhanced and new activities, noting that such an approach needs high-resolution structures of an enzyme’s active site to be successful, preferably with substrates bound. In contrast to previous reports that have aimed to widen the catalytic activity and stereospecificity of highly stereoselective enzymes, our study has employed a stereochemically promiscuous aldolase enzyme as a template to develop stereoselective variants,^30^ using structurally informed site-directed mutagenesis to generate a suite of mutant aldolases that can be used to prepare all four possible aldol stereoisomers. Structural determination of the variant enzymes has allowed us to rationalize many of the observed changes in catalytic activity and stereoselectivity, with simulations indicating that stereoselectivity likely arises during carbon–carbon bond formation. Notably, some of the mechanisms that produce the observed structural changes were not as originally predicted, once again demonstrating the challenge of modifying enzyme stereoselectivity through a structure-guided approach.

## Supporting Information Available

Parameters for the pyruvate-lysine Schiff base (KPI) used in the molecular dynamics simulations (ZIP)
HPLC traces for aldolase-catalyzed reactions of Variants 3 and 4, images of the connected density and bound product in Variant 1, of the bound product in Variant 2, and around the bound substrate in Variant 3, table of crystallographic data, details of the enzyme–substrate molecular dynamics simulations, synthetic procedures, characterization data for intermediates and products, and relevant NMR spectra (PDF)

## Abbreviations

SsKDG-aldolase: *Sulfolobus solfataricus* 2-keto, 3-deoxygluconate aldolase
d-KDGlc: d-2-keto-3-deoxy gluconate
d-KDGal: d-2-keto-3-deoxy galactonate
l-KDGlc: l-2-keto-3-deoxy gluconate
l-KDGal: l-2-keto-3-deoxy galactonate.
