# Supplementary material for: Structurally Informed Mutagenesis of a Stereochemically Promiscuous Aldolase Produces Mutants That Catalyze the Diastereoselective Syntheses of All Four Stereoisomers of 3-Deoxy-hexulosonic Acid
Source: ACS Catal. 2022 Sep 6;12(18):11444–55. doi: 10.1021/acscatal.2c03285 (PMC9486944; doi:10.1021/acscatal.2c03285)
Supplement: Supplementary file 2 — cs2c03285_si_002.pdf [file cs2c03285_si_002.pdf]

Supporting Information

Structurally Informed Mutagenesis of a Stereochemically Promiscuous Aldolase Produces Mutants That Catalyse the Diasteroselective Syntheses of All Four Stereoisomers of 3-Deoxy-hexulosonic Acid

Sylvain F. Royer[1,4], Xuan Gao[2], Robin Groleau[3], Marc W. van der Kamp[2], Steven D. Bull[3], Michael J. Danson[1] and Susan J. Crennell[1*]

[1]Department of Biology and Biochemistry, University of Bath, Bath, BA2 7AY, UK

[2]School of Biochemistry, University of Bristol, University Walk, Bristol, BS8 1TD, UK

[3]Department of Chemistry, University of Bath, Bath, BA2 7AY, UK

[4]Present address: University of Oxford, Chemistry Research Laboratory, Mansfield Road, Oxford, OX1 3TA, UK

Corresponding author email address: S.J.Crennell@bath.ac.uk

ASSOCIATED CONTENT

Contents of the supporting information:

1. HPLC analysis of aldolase-catalysed reactions 2
2. Supplementary structure images and X-ray data collection and model refinement statistics 4
3. Enzyme-substrate molecular dynamics simulations 7
4. Synthetic procedures and characterization data for intermediates and products. 11
5. ^{1}H and ^{13}C NMR spectra of D-KDGlc and L-KDGal 17
6. References 22

1. HPLC analysis of aldolase-catalysed reactions

Figure S1. Selected HPLC traces for aldolase-catalyzed reactions of pyruvate and L-glyceraldehyde

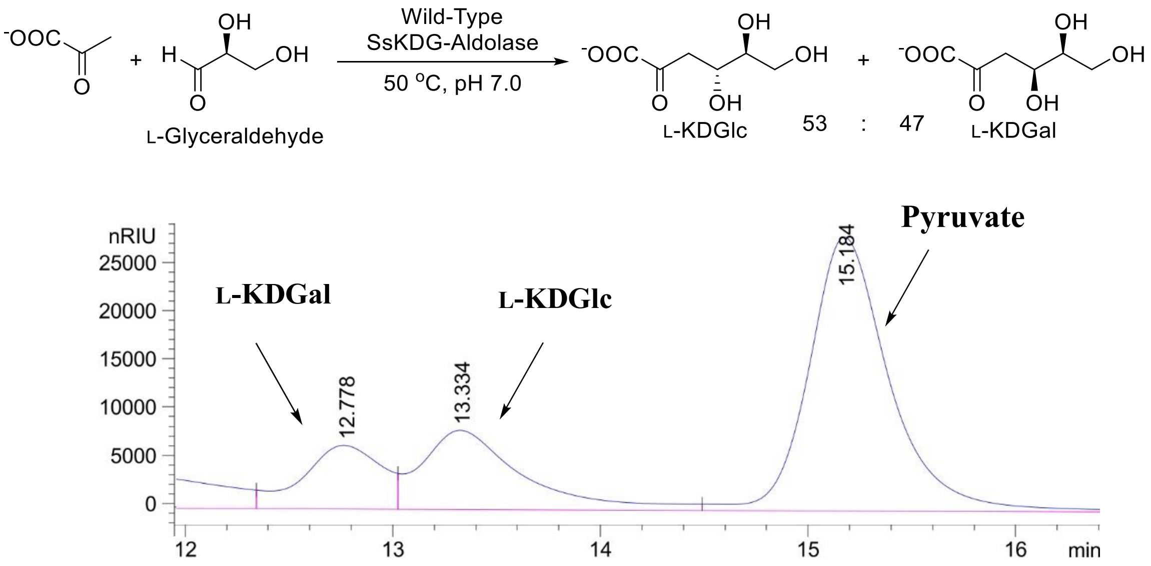

(a) HPLC trace of non-selective aldol reaction of L-glyceraldehyde catalyzed by wild-type KDG-aldolase

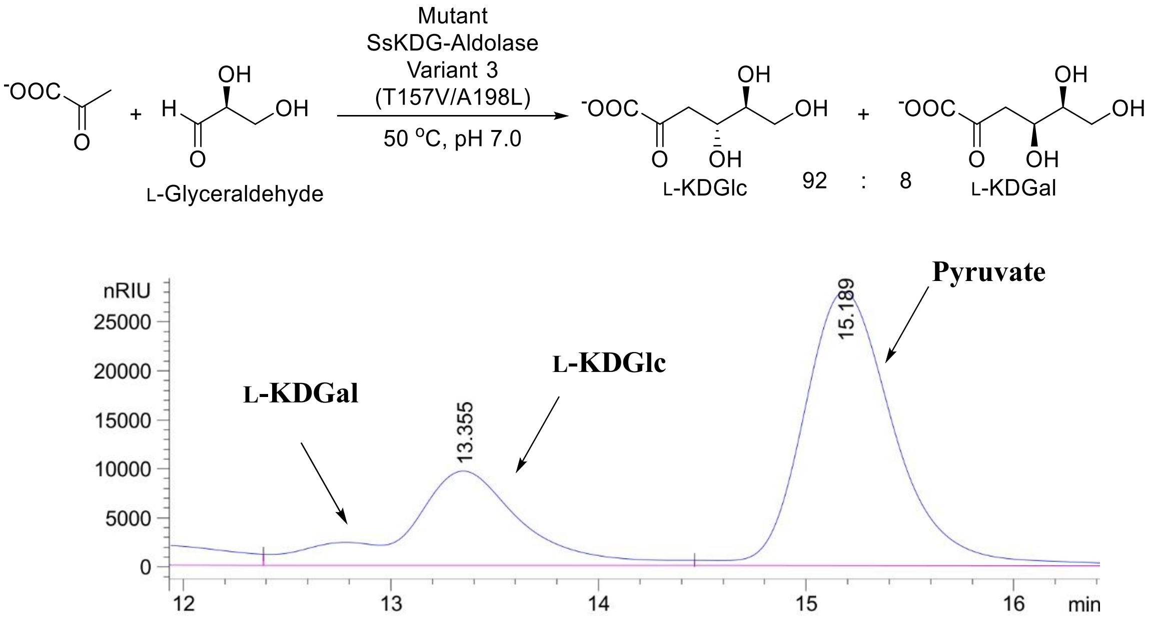

(b) HPLC trace of aldol reaction of L-KDGlc selective double mutant Thr-157-Val | Ala-198-Lys (variant 3)

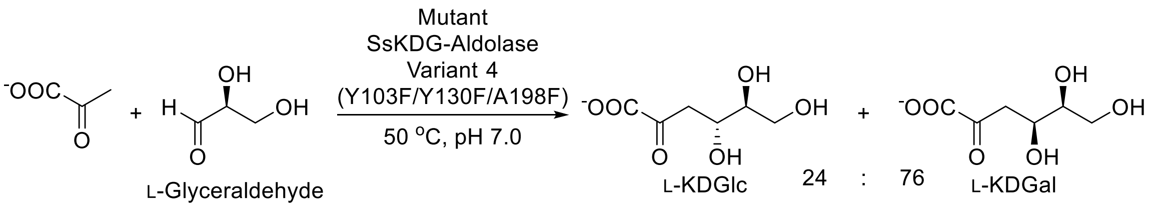

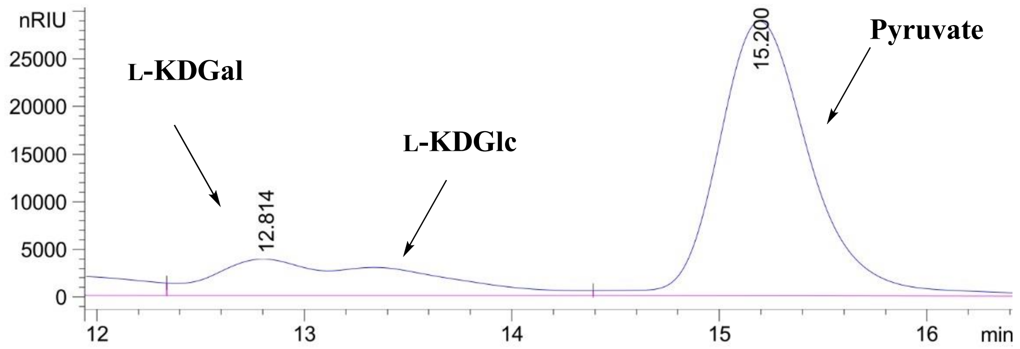

(c) HPLC trace of aldol reaction of L-KDGal selective triple mutant Tyr-103-Phe│ Tyr–130–Phe │ Ala–198–Phe (variant 4)

HPLC traces for aldol reactions catalysed by D-KDGlc selective variant 1 (Tyr-132-Val │ Thr-157-Cys) and D-KDGal selective variant 2 (Thr-157-Val │ Asp-181-Gln │ Ala–198-Lys) are reported in the Supporting Information of Reference 1.

2. Supplementary structure images and X-ray data collection and model refinement statistics

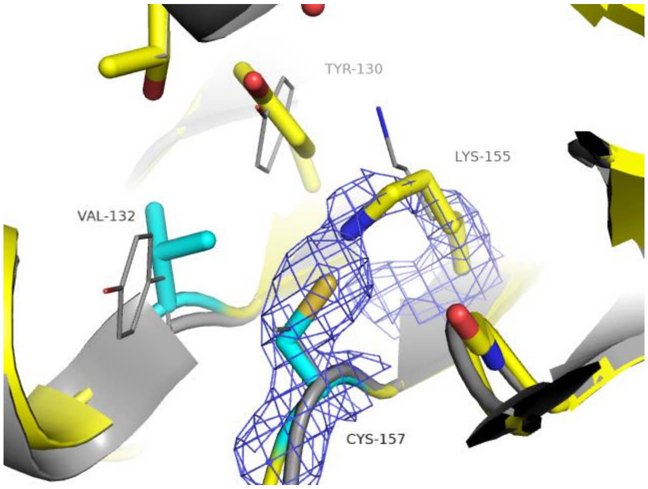

Figure S2. Alignment of the active site of Variant 1 (unsoaked, yellow, with mutated residues in cyan) with the active site of the wild-type enzyme (PDB code: 1W37, grey cartoon and amino acid lines). A 2FoFc electron density map (contoured at 1.5σ) is shown around amino acids 155 and 157 of Variant 1.

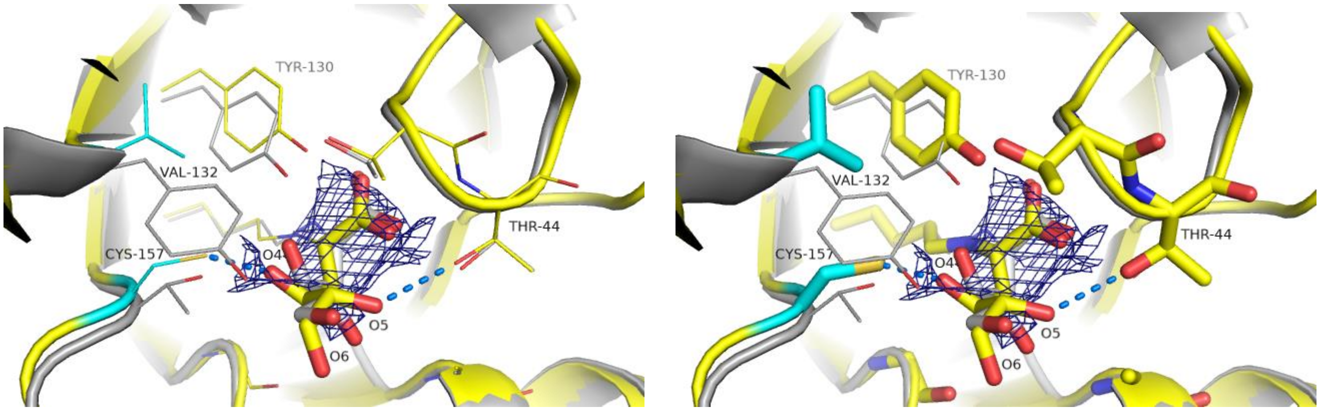

Figure S3 The active site of Variant 1 in complex with D-KDGlc superimposed onto the corresponding D-KDGlc complex of the wild-type enzyme. Variant 1 amino acids are shown as yellow sticks, with mutations shown in cyan. Conformations of D-KDGlc in wild-type (grey) and Variant 1 (yellow) complexes are shown, together with stabilising hydrogen bond interactions (blue dotted lines) between O4 and O5 of D-KDGlc and residues in the active site. 2FoFc electron density map (contoured at 1σ) around the D-KDGlc is shown in grey lines.

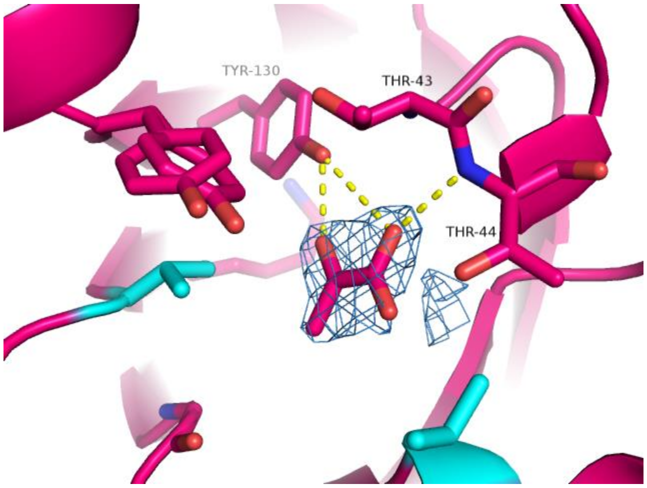

Figure S4. Active site of Variant 2 co-crystallised with pyruvate, showing the quality of the 2FoFc map around pyruvate (contoured at 1σ). Hydrogen bonds made with the protein are shown as yellow dotted lines. The side chain of Y132 occupies 2 conformations in the absence of substrate interaction.

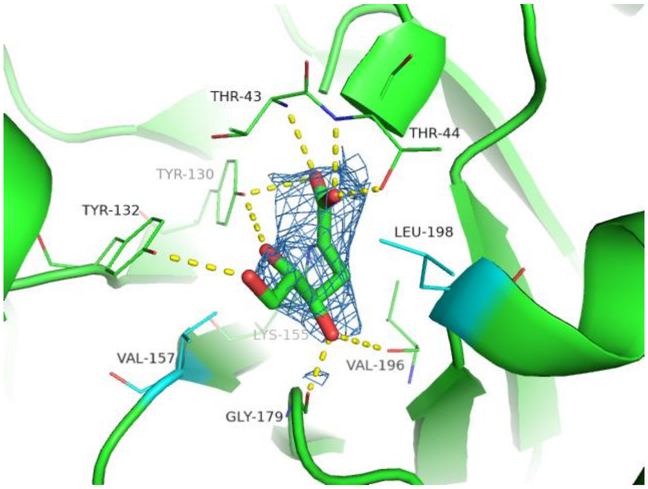

Figure S5. Active site structure of Variant 3 obtained from a 2-minute soak in L-KDGlc. Active site residues are drawn as sticks and labelled, with mutated amino acids shown in cyan. Polar contacts between L-KDGlc and the enzyme are shown as yellow dotted lines. The 2FoFc map (contoured at 1σ) around the L-KDGlc ligand is shown in mesh representation.

Table S1. X-ray data collection, processing and model refinement parameters for SsKDG-aldolase variants. Values in parentheses are for the highest resolution data shell.

	**Variant 1**		**Variant 2**		**Variant 3**		**Variant 4**	
Specificity	D-KDGlc:D-KDGal 93:07		D-KDGlc:D-KDGal 12:88		L-KDGlc:L-KDGal 92:08		L-KDGlc:L-KDGal 24:76	
Crystallisation	15%PEG4K, 8%IPA, pH 6.0		11% PEG4K, 4% IPA, pH 6.1		13% PEG4K, 8% IPA, pH 6.8		15% PEG4K, 12% IPA, pH 5.9	
Ligand/soak time	-	D-KDGlc/24 h	D-KDGal/2 min	co-crystallised	L-KDGlc/45 s	L-KDGlc/2 min	-	L-DKGal/1 min
Ligand bound	-	D-KDGlc & pyruvate	-	Pyruvate	-	L-KDGlc	-	L-KDGal & pyruvate
Data source	Diamond i04	in-house	Diamond i04	in-house	in-house	in-house	Diamond i03	Diamond i03
Merging software	Dials	Mosflm	Mosflm	HKL2000	HKL2000	HKL2000	Xia2	HKL2000
Resolution (Å)	2.0(2.03-2.0)	3.15(3.37-3.15)	1.57(1.60-1.57)	2.17(2.21-2.17)	2.8(2.85-2.80)	3.2(3.26-3.2)	1.91 (1.96-1.91)	2.1(2.14-2.1)
Space group	$P2_12_12_1$	$P6_522$	$P2_1$	$P2_1$	$P2_12_12_1$	$P6_522$	$P6_522$	$P6_522$
Unit cell (Å)	57.0 144.6 161.5	a=b=103.1, c=243.4	76.4 86.9 92.0 β=95.8°	76.5 87.0 92.0 β=95.7°	83.1 126.0 126.8	a=b=105.1, c=246.4	a=b=99.62 c=249.01	a=b=103.5 c=245.6
Rmerge	0.050(0.377)	0.090(0.275)	0.047(0.361)	0.064(0.225)	0.134(0.517)	0.201(0.593)	0.104(0.69)	.087(0.605)
Completeness (%)	99.4(100.0)	95.2(82.0)	97.9(88.5)	95.3(79.1)	89.3(87.3)	95.4(99.9)	99.6(97.7)	99.9 (100)
I/sig(I)	10.4(2.1)	22.0 (5.3)	12.6(2.1)	23.3(4.59)	7.95(1.87)	5.7(2.30)	16.1(2.7)	22.2(3.06)
Observed	288023 (14310)	169852(12593)	595658(22370)	782100	618110	229129	589108(29282)	2188043
Unique	90479(4466)	13200(1959)	161665(7218)	60596	30037	13375	57132(4059)	46234
Multiplicity	3.2(3.2)	12.9(6.4)	3.7(3.1)	5.4(2.6)	2.9(2.2)	2.6(2.8)	10.3(7.2)	6.8(6.5)
Refinement software	Phenix	Phenix	Phenix	Phenix	Phenix	Phenix	Refmac	Phenix
R	0.1817	0.2005	0.1477	0.145	0.1873	0.1775	0.1700	0.1643
Rfree	0.2357	0.2363	0.1750	0.195	0.2338	0.2440	0.2104	0.2124
RMSD bond lengths	0.012	0.009	0.008	0.011	0.004	0.003	0.016	0.008
RMSD bond angles	1.069	1.260	0.852	1.054	0.664	0.630	1.42	0.89
Molprobity score	1.65	1.92	1.39	1.17	1.44	1.65	1.29	1.27
PDB code	6GSO	6GT8	6H2R	6H2S	6H7R	6H7S	2YDA	6GV2

3. Enzyme-substrate molecular dynamics simulations

Preparation of protein and substrate coordinates

Molecular dynamics (MD) simulations were performed to model the state in which there is a pyruvate-lysine Schiff base (KPI) together with a (non-covalently bound) glyceraldehyde (D- or L-). In total, 12 systems of SsKDG-aldolase variants were prepared, based on X-ray data as outlined in Table S2 (see Table S1 for crystal structure details). WT SsKDG-aldolase and variants 1 and 2 were prepared in complex with D-glyceraldehyde, either in position to form D-KDGlc or to form D-KDGal. WT SsKDG-aldolase and variants 3 and 4 were prepared in complex with L-glyceraldehyde, either in position to form L-KDGlc or to form L-KDGal. Coordinates of variants with high resolution crystal structures were selected and assembled with appropriate substrate coordinates using PyMOL (The PyMOL Molecular Graphics System, Version 2.3.0, Schrödinger, LLC.). The simulation setup of 12 coordinates is shown in Table S2 which indicates coordinate sources of SsKDG-aldolase and substrates. Note that two crystal structures of Variant 3 are low in resolution (Table S1); the setup of Variant 3 is therefore based on the Variant 2 structures, because they differ by only a single mutation (Q/D181).

Table S2. Simulation setup of protein and substrate coordinates and residue numbers of histidine tautomers.

	Protein coord.	Substrate coord.	HID	HIE	HIP
Wild-type D-KDGlc	1W3N	1W3N	N/A	A: 26,111,229<br>B: 26,111,125,229<br>C: 26,111,125,229<br>D: 26,111,125,229	A: 125,163<br>B: 163<br>C: 163<br>D: 163
Wild-type D-KDGal	1W3N	1W3T			
Variant 1 D-KDGlc	6GSO	1W3N	N/A	A: 26,111,125,229<br>B: 26,111,125,229<br>C: 26,111,229<br>D: 26,111,125,229	A: 163<br>B: 163<br>C: 125,163<br>D: 163
Variant 1 D-KDGal	6GSO	1W3T			
Variant 2 D-KDGlc	6H2R	1W3N	A: 111,229<br>B: 111,229<br>C: 229<br>D: 111,229	A: 125<br>B: N/A<br>C: 26,111<br>D: 125	A: 26,163<br>B: 26,125,163<br>C: 125,163<br>D: 163
Variant 2 D-KDGal	6H2R	1W3T			
Wild-type L-KDGlc	1W3N	6H7S	N/A	A: 26,111,229<br>B: 26,111,125,229<br>C: 26,111,125,229<br>D: 26,111,125,229	A: 125,163<br>B: 163<br>C: 163<br>D: 163
Wild-type L-KDGal	1W3N	6GV2			
Variant 3 L-KDGlc	6H2R*	6H7S	A: 111,229<br>B: 111<br>C: 229<br>D: 111,229	A: 125<br>B: 229<br>C: 26,111<br>D: 125	A: 26,163<br>B: 26,125,163<br>C: 125,163<br>D: 163
Variant 3 L-KDGal	6H2R*	6GV2			
Variant 4 L-KDGlc	6GV2	6H7S	A: 111<br>B: 111<br>C: 111<br>D: 111	A: 125,229<br>B: 26,125,229<br>C: 26,125,229<br>D: 26,125,229	A: 26,163<br>B: 163<br>C: 163<br>D: 163
Variant 4 L-KDGal	6GV2	6GV2			

*Q181 mutated back to D181 using PyMOL.

Molecular dynamics setup and simulations

The glyceraldehyde was only modelled in chain A of the tetramer in all systems. Water molecules from SsKDG-aldolase structures were kept. The Enlighten2 PREP protocol (see: https://github.com/vanderkamp/enlighten2) was performed to generate Amber topology and coordinate files for further MD simulations.[2] This involves addition of hydrogens to the protein using AmberTools18 programs pdb4amber and reduce.[3] Protonation states of titratable residues were predicted by PropKa 3.1.[4,5] Histidine tautomers (predicted by reduce) were manually edited to ensure consistency within variants (see Table S2). All ionizable residues were in their standard protonation states (Asp and Glu negatively charged, Lys and Arg positively charged). A 30 Å solvent sphere was added by the AmberTools18[3] program tleap, centered on the carbon atom of pyruvate which forms a carbon-carbon bond with D- or L-glyceraldehyde. The ff14SB force field was used to treat the protein and the TIP3P model was used for water.[6] AmberTools18 programs antechamber, prmchk2 and sqm are used for parameterization of the ligand with the General Amber Force Field version 2 (GAFF2).[2,7] Parametrization for the pyruvate-lysine Schiff base (KPI) was performed using the RED server (https://upjv.q4md-forcefieldtools.org/)[9], with partial charges obtained from restrained electrostatic potential (RESP) fitting and missing force field parameters taken from analogous GAFF parameters. Parameter files for KPI are available as Supporting Information files (KPI.off and KPI.frcmod).

After preparation of the systems, the following steps were performed using the AmberTools program sander to relax the structures:

1) brief energy minimization of all hydrogens (with positional restraints of 50 kcal mol^{-1} Å^{-2} on all other atoms);
2) brief energy minimization of hydrogens within 22 Å of the solvent sphere center;
3) energy minimization of all atoms within 22 Å of the solvent sphere center.

After this minimization protocol, the carbon-carbon distance between glyceraldehyde and KPI was consistent with a 'near attack' or 'reaction ready' pose (between 2.8 and 3.4 Å).

MD simulations were based on the Enlighten2 dynamics protocol.[2] Prior to the 'production' MD simulations, a 5 ps heating process to 300 K and subsequently 50 ps dynamics were performed with carbon-carbon distance restraint (3.5 Å, force constant: 100 kcal mol^{-1} Å^{-2}). Then, production simulations (100 ps, 1000 snapshots) were performed without distance restraint. During simulation, atoms within 26 Å of the solvent sphere center were free to move, SHAKE was applied to bonds containing hydrogens and a 2 fs timestep was employed. 10 independent sets of simulations (heating, 50 ps MD with carbon-carbon distance restraint, 100 ps without restraint) were run for all 12 variant systems, and the resulting trajectories analyzed with the AmberTools18 program CPPTRAJ.[8] The percentage of 'reactive' poses (for formation of KDGlc or KDGal) was calculated from the trajectory based on the following geometric criteria: (1) the expected stereoisomer (KDGlc/KDGal) formed was 'predicted' by the approach of the aldol-group to the pyruvate (KPI) carbon, expressed as a dihedral angle between the plane of the aldol group (carbon, oxygen and hydrogen) and the plane formed by the pyruvate carbon, aldol carbon and aldol oxygen (Figure S6, D-glyceraldehyde as the starting structure). (2) the distance between carbon (pyruvate of

KPI) and carbon (substrate) indicates if the carbon-carbon bond is ready to be formed, with a cut-off value of less than 4.0 Å (shorter cut-off distances give similar results).

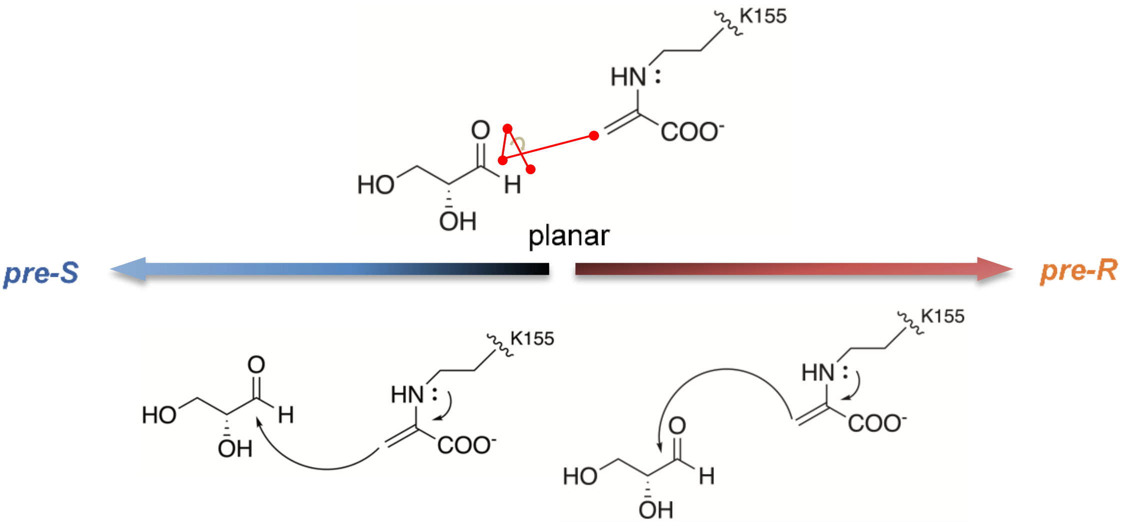

Figure S6. Dihedral angle used to indicate pre-*S*/pre-*R* poses. The dihedral angle is measured between the plane formed by the pyruvate carbon, aldol carbon and aldol oxygen and the plane of the aldol group (carbon, oxygen and hydrogen). Taking the D-glyceraldehyde as the starting structure, a dihedral angle between −180° and 0° favors pre-*R* (D-KDGal) geometry, whilst a dihedral angle between 0° and 180° has preference for pre-*S* (D-KDGlc) geometry. The positive and negative ranges of dihedral angles for the expected stereoisomer are the opposite for L-glyceraldehyde starting structure.

Molecular dynamics simulations results

The orientation of reactive poses from all MD simulations as calculated based on the above criteria is shown in Figure S7. Variants in Figure S7 (a) (WT, Variant 1 and Variant 2) take D-glyceraldehyde as the substrate, and can form D-KDGlc or D-KDGal. According to the glyceraldehyde complex simulations, these three SsKDG-aldolase variants show a clear preference for binding modes leading to D-KDGlc. This is not consistent with the preference indicated by the enzyme assay results (see Figure 1 (a), Figure 3 (a) and (b)). Similarly, the simulations with L-glyceraldehyde (for WT, Variant 3 and Variant 4, Figure S7 (b)), indicate a preference for binding modes leading to L-KDGal. This again is not consistent with the experimental stereoselectively (Figure 1 (b), Figure 3 (c) and (d)). Overall, this indicates that the glyceraldehyde binding orientation is not the main contributing factor to the observed stereoselectivity. (Classification of pre-*R*/D-KDGal/L-KDGlc or pre-*S*/D-KDGlc/L-KDGlc is based here on the positive or negative dihedral angle formed by pyruvate carbon and aldol group of glyceraldehyde. Many snapshots display dihedral angles that do not allow a clear distinction between KDGlc or KDGal – *e.g.* values near 0°. However, results remain very similar when only those snapshots are considered with dihedral angles between −135° to −45° and between 45° to 135°.)

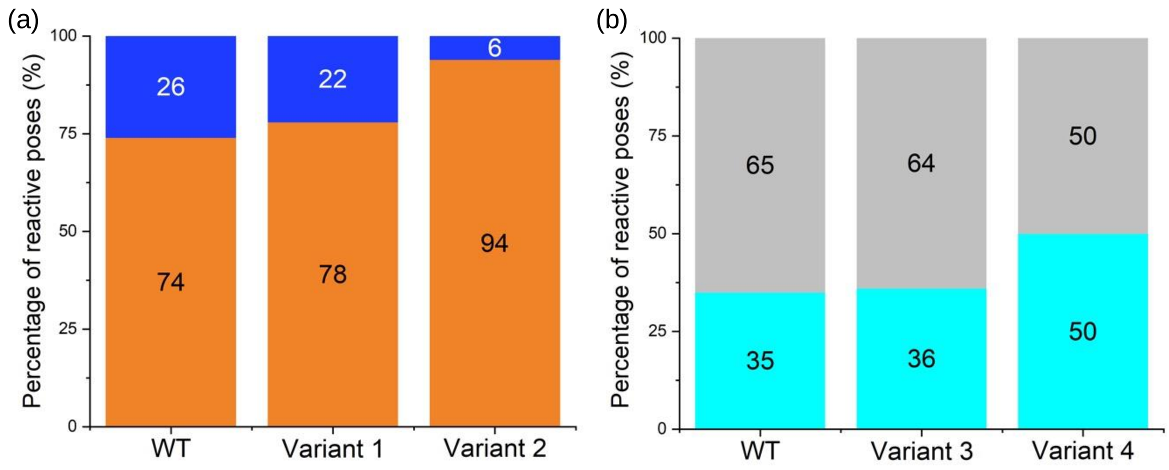

Figure S7. Percentage of 'reaction ready' poses (C-C distance cut-off value: 4 Å) of variants with different starting structures. (a) wild-type, Variant 1 and Variant 2 with D-glyceraldehyde as the starting structure (Blue: glyceraldehyde positioned to form D-KDGal, Orange: glyceraldehyde positioned to form D-KDGlc). (b) wild-type, Variant 3 and Variant 4 with L-glyceraldehyde as the starting structure (Grey: glyceraldehyde positioned to form L-KDGal, Cyan: glyceraldehyde positioned to form L-KDGlc).

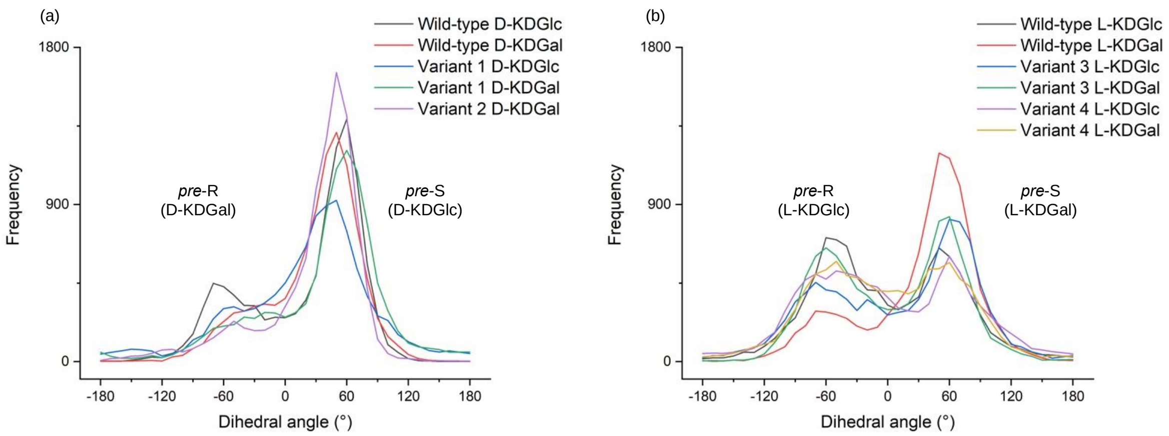

Figure S8. Histograms of dihedral angle of variants with different starting structures. (a) D-glyceraldehyde and (b) L-glyceraldehyde. The lines are labeled with the starting models used for the simulation, as detailed in Table S2. V2 D-KDGlc is not shown due to its high preference for D-KDGlc conformation (high peak at pre-*S*, around 60°).

4. Synthetic procedures and characterization data for intermediates and products.

4.1. General experimental details

Reagents and solvents were obtained from commercial suppliers and used without further purification. Reactions were performed without air exclusion or drying, at room temperature, with magnetic stirring, unless otherwise stated. Anhydrous Na_2SO_4 was used as drying agents for organic solvents.

Capillary melting points were determined using a Stuart digital SMP10 melting point apparatus and are reported uncorrected to the nearest °C. Optical rotations were measured using an Optical Activity Ltd AA-10 Series Automatic Polarimeter, with a path length of 1 dm, and with concentration (*c*) quoted in g/100 mL.

Nuclear Magnetic Resonance (NMR) spectroscopy experiments were performed in deuterated solvent at 298 K (unless stated otherwise) on an Agilent ProPulse 500 MHz spectrometer. ^1H and ^{13}C chemical shifts (δ) are quoted in parts per million (ppm) and are referenced to either the residual solvent peak or tetramethylsilane (TMS) when possible. Coupling constants (J) are quoted in Hz.

Infrared (IR) spectra were recorded using a PerkinElmer Spectrum 100 FTIR spectrometer fitted with a Universal ATR FTIR accessory, with samples run neat and the most relevant, characteristic absorbances quoted as ν in cm^{-1}.

High resolution mass spectrometry (HRMS) results were acquired on an externally calibrated Agilent QTOF 6545 with Jetstream ESI. Molecular ions were detected in negative ionisation mode as deprotonated/desodiated species.

4.2. Synthesis and characterization of D-glyceraldehyde

1,2-5,6-Di-*O*-isopropylidene-D-mannitol

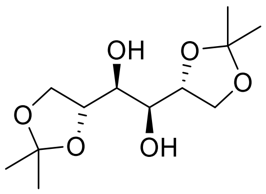

Following the method of De Alvarenga *et al.*:[10] $ZnCl_2$ (20.0 g, 146 mmol, 2.66 equiv.) was dissolved in acetone (anhydrous, 200 mL) under an N_2 atmosphere at room temperature. D-Mannitol (10.0 g, 54.8 mmol, 1.00 equiv.) was added to this solution in one batch, and the reaction allowed to stir at room temperature until full dissolution (5 h). After this time, the reaction was cooled to 0 °C, a suspension of $NaHCO_3$ (20 g) in water (20 mL) was added, and the mixture stirred vigorously for 15 min. The mixture was filtered to remove the precipitated zinc carbonate, which was washed with acetone (20 mL), and the crude product concentrated to dryness *in vacuo*. The crude product was dissolved in Et_2O and water (25 mL each), separated, and the aqueous phase was extracted twice more with Et_2O (2 x 25 mL). The combined organics were then washed with brine (50 mL), dried over Na_2SO_4, and concentrated to dryness in vacuo. The crude product was

triturated with *n*-hexane to afford 1,2-5,6-di-*O*-isopropylidene-D-mannitol (7.085 g, 26.9 mmol) as a white solid in 49% yield. Characterization data were consistent with previous literature reports.[10,11]

$[\alpha]_D^{23}$= +7 ($CHCl_3$, *c* = 1.0) (lit.[11] $[\alpha]_D^{25}$ = +7.0, $CHCl_3$, *c* = 1.0); m.p.: 115-117 °C (lit.[11] 115-118 °C); IR (neat): *ν* 3392, 3283, 2979, 2930, 2891, 1385, 1371, 1260, 1205, 1157, 1062, 1039, 1003, 944, 857, 651 cm^{-1}; ^{1}H NMR (500 MHz, $CDCl_3$) δ_H 4.20 (m, *J* = 6.4 Hz, 2 x OCH_2CH), 4.12 (dd, 2H, *J* = 8.6, 6.3 Hz, 2 x ½ CH_2), 3.97 (dd, 2H, *J* = 8.6, 5.7 Hz, 2 x ½ CH_2), 3.75 (t, 2H, *J* = 6.5, 6.4 Hz, 2 x HOC*H*), 2.55 (d, 2H, *J* = 6.5 Hz, 2 x OH), 1.42 (s, 6H, 2 x CH_3), 1.36 (s, 6H, 2 x CH_3); ^{13}C{^{1}H} NMR (126 MHz, $CDCl_3$) δ_C 109.5, 76.5, 71.4, 66.9, 26.9, 25.3.

D-Glyceraldehyde acetonide.

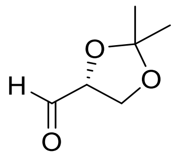

Following the method of Ferrié *et al.*:[12] Saturated aqueous $NaHCO_3$ (0.5 mL) was added to a solution of compound 1,2-5,6-di-*O*-isopropylidene-D-mannitol (1.049 g, 4.0 mmol, 1.0 equiv,) in DCM (10 mL). $NaIO_4$ (1.711 g, 8.0 mmol) was then added in 5 batches over 10 min, and the mixture stirred for 1 h at room temperature. After this time, Celite (1.0 g) and $MgSO_4$ (0.5 g) were added, and the mixture stirred for a further 15 min. The reaction was then filtered, and the solvent removed *in vacuo* at room temperature. The residue was purified by bulb-to-bulb distillation at atmospheric pressure under N_2 to yield D-glyceraldehyde acetonide (0.931 g, 7.12 mmol) as a colourless oil in 89% yield. This intermediate could also be purified by column chromatography (SiO_2, 30% EtOAc *n*-hexane/EtOAc). Characterization data were consistent with previous literature reports.[12,13]

This intermediate was used immediately due to its low stability to avoid degradation and polymerization. If stored at -18 °C for extended periods, redistillation is required in order to crack the reagent prior to use.

R_f = 0.21 (SiO_2, 30% EtOAc/*n*-hexane); $[\alpha]_D^{29}$ = +38 ($CHCl_3$, *c* = 1.0) (lit.[13] $[\alpha]_D$ = +40, $CHCl_3$, *c* = 1.0); ^{1}H NMR (500 MHz, $CDCl_3$) δ_H; 9.72 (d, 1H, *J* = 2.0 Hz, C(O)*H*), 4.38 (ddd, 1H, *J* = 6.8, 4.8, 2.0, HC(O)C*H*), 4.22-4.06 (m, 2H, CH_2), 1.49 (s, 3H, CH_3), 1.42 (s, 3H, CH_3); ^{13}C{^{1}H} NMR (126 MHz, $CDCl_3$) δ_C 201.9, 111.4, 80.0, 65.7, 26.4, 25.3.

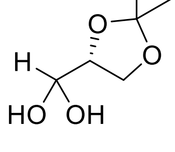

D-Glyceraldehyde acetonide was also observed as the hydrate *gem*-diol by NMR spectroscopy in D_2O: ^{1}H NMR (500 MHz, D_2O) δ_H; 5.02-4.93 (m, 1H, $(HO)_2CH$), 4.23-3.92 (m, 3H, CH_2CH), 1.48 (s, 3H, CH_3), 1.42 (s, 3H, CH_3); ^{13}C{^{1}H} NMR (126 MHz, D_2O) δ_C 110.4, 90.2, 77.6, 65.2, 25.4, 24.0.

D-Glyceraldehyde.

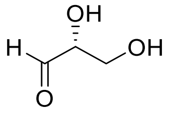

D-Glyceraldehyde acetonide (1.0 equiv.) was added to a mixture of TFA/water (80 mM, pH 1-1.5, 20 mL/mmol of substrate). After stirring for 1.5 h at room temperature, the mixture was concentrated *in vacuo* to afford D-glyceraldehyde as an oily film in quantitative yield. This product was used immediately due to its low stability, to avoid degradation and polymerization.

$[\alpha]_D^{25}$ = +9 (*c* = 2.0, H_2O), Lit.[14] $[\alpha]_D^{22}$ = +9.75 (*c* = 2.0, H_2O)

4.3. Synthesis and characterization of L-glyceraldehyde

L-Glyceraldehyde acetonide.

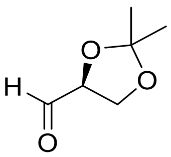

Following the method of Stecko *et al.*:[15] Methyl (*S*)-2,3-*O*-isopropylidenepropionate (2.402 g, 15.0 mmol, 1.0 equiv.) was dissolved in anhydrous DCM (20 mL) under an N_2 atmosphere, and cooled to -78 °C. To this, a solution of DIBAL-H (1.0 M in hexanes, 16.5 mL, 15.6 mmol, 1.1 equiv.) was added dropwise over 15 min, and the reaction then allowed to stir for 3 h (completion monitored by TLC, 3:1 hexanes/EtOAc, $KMnO_4$ stain). After this time, the reaction mixture was diluted with Et_2O (35 mL), and 0.64 mL of water added dropwise over 5 min. The reaction was then stirred vigorously and warmed to 0 °C, and 1.0 mL of 4.0 M NaOH added. After a further 5 min of vigorous stirring, another 1.5 mL of water was added to the reaction, followed by warming to room temperature and stirring for 15 min. The reaction mixture was then dried using $MgSO_4$, filtered, and solvents were removed *in vacuo*. The residue was purified by bulb-to-bulb distillation at atmospheric pressure under N_2 to yield D-glyceraldehyde acetonide (1.640 g, 12.6 mmol) as a colourless oil in 68% yield. This intermediate could also be purified by column chromatography (SiO_2, 30% EtOAc *n*-hexane/EtOAc). Characterization data were consistent with previous literature reports.[10,11]

This intermediate was used immediately due to its low stability to avoid degradation and polymerization. If stored at -18 °C for extended periods, redistillation is required in order to crack the reagent prior to use.

R_f = 0.21 (SiO_2, 30% EtOAc/*n*-hexane); $[\alpha]_D^{29}$= -37 ($CHCl_3$, *c* = 1.0) (lit.[13] $[\alpha]_D$ = +40 for D-enantiomer, $CHCl_3$, *c* = 1.0); ^{1}H NMR (500 MHz, $CDCl_3$) δ_H 9.71 (d, 1H, *J* = 2.0 Hz, C(O)*H*), 4.55-3.62 (m, 3H, C*H*+C*H*$_2$), 1.44 (s, 3H, C*H*$_3$), 1.38 (s, 3H, C*H*$_3$); ^{13}C{^{1}H} NMR (126 MHz, $CDCl_3$) δ_C 201.9, 111.4, 80.0, 65.7, 26.4, 25.3.

L-Glyceraldehyde.

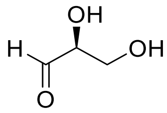

L-glyceraldehyde was prepared via acid catalysed hydrolysis L-glyceraldehyde acetonide as described previously for formation of D-glyceraldehyde (see section 4.2). This product was used immediately due to its low stability to avoid degradation and polymerization.

$[\alpha]_D^{25}$ = -9 (c = 2.0, H_2O), Lit.[14] $[\alpha]_D^{22}$ = -9.17 (c = 2.0, H_2O)

4.4. Synthesis and characterization of KDGal and KDGlc stereoisomers

All four KDGal/KDGlc stereoisomers were individually prepared in >95:5 dr using the appropriate variant aldolase to catalyse aldol reactions between D- or L-glyceraldehyde using the preparative/purification procedures described in the Methods and Materials section of the main article. Structural characterization of the KDGlc and KDGal products was hindered by the lack of high quality $^1H/^{13}C$ NMR spectroscopic characterization data reported for these compounds in the literature. The $^1H/^{13}C$ NMR spectra of KDGlc and KDGal are complicated by: (a) The presence of multiple interconverting open-chain, α-/β-pyranose and α-/β-furanose species in aqueous solution (shown for KDGlc in Scheme S1a); (b) The potential of the keto groups of KDGlc and KDGal to undergo keto-enol tautomerism (shown for KDGlc in Scheme 1b); (c) Hydration of the keto groups of KDGlc and KDGal to form gem-diols (shown for KDGlc in Scheme 1c).[16] The ratio of interconverting KDGal/KDGlc species present in aqueous solution are also dependent on concentration, pH and the presence of any impurities, which combine to complicate NMR analysis of mixtures of KDGlc/KDGal further. Consequently, NMR analysis was not used to determine dr values for KDGlc/KDGal formation in these aldolase catalyzed reactions, with aldol product ratios instead more accurately determined by HPLC analysis as reported previously (*vide supra*).[14,17]

a)
b)
c)

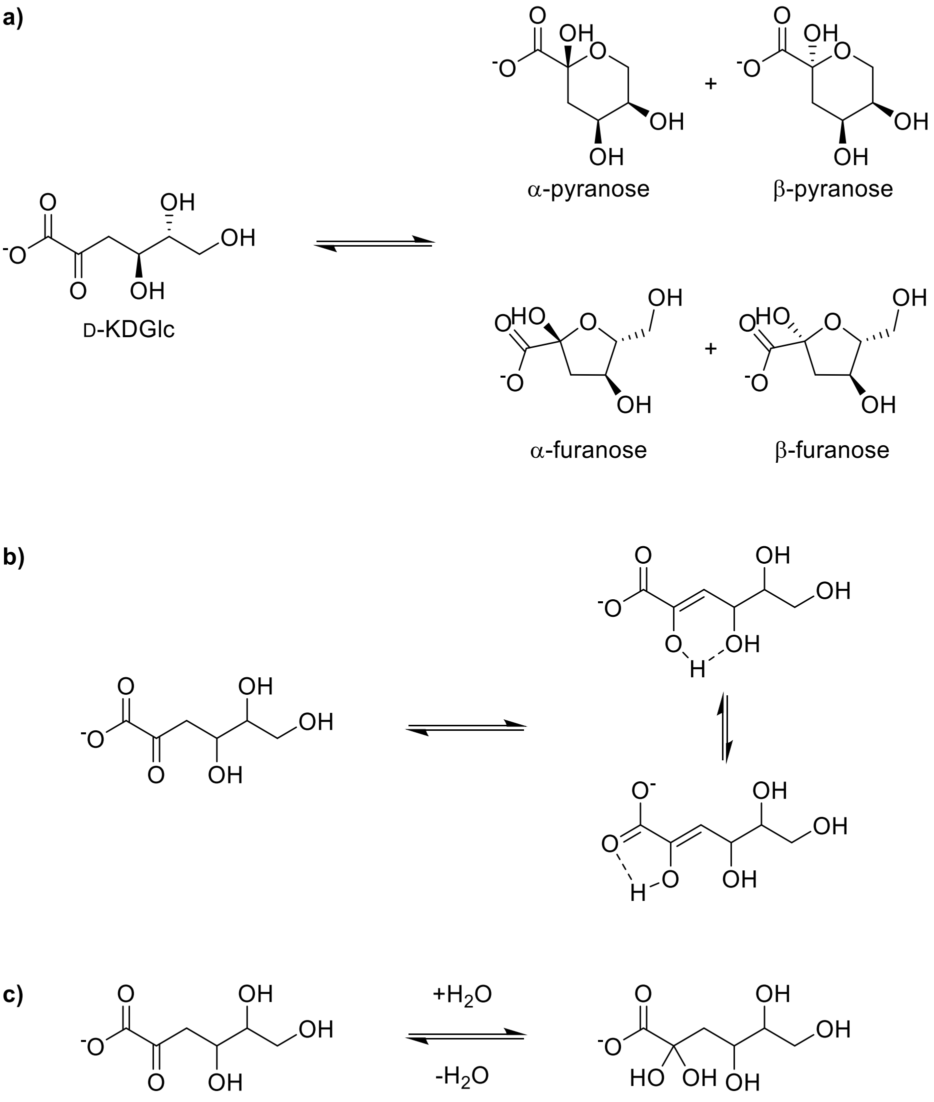

Scheme S1: Potential interconverting KDGlc species that are present in aqueous solution. (a) Interconverting open-chain and α-/β-furanose and α-/β-pyranose forms. (b) Keto-enol tautomerization of the α-keto acid fragment. (c) Hydration of the α-keto acid fragment to produce its corresponding hydrate.

Representative characterization data for KDGlc and KDGal:

D-KDGlc.

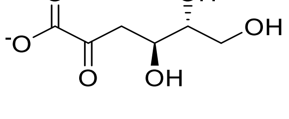

D-KDGlc was produced from D-glyceraldehyde using variant 1 $[\alpha]_D^{26}$= -31.5 (H_2O, *c* = 1.3) (lit.[18] $[\alpha]_D^{25}$ = -34.1, H_2O, *c* = 1.3); Images of ^1H and ^{13}C NMR spectra reported in Section 5; IR (neat): *ν* 3302, 2947, 2466, 2362, 2015, 1978, 1716, 1667, 1441, 1369, 1190, 1134,

1033, 841, 799, 724 cm^{-1}; HRMS (ESI–): Calculated for $C_6H_9O_6^-$: 177.0394; Found: 177.0405.

L-KDGal.

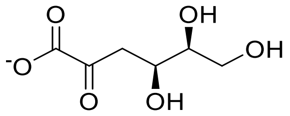

L-KDGal was produced from L-glyceraldehyde using variant 4. $[\alpha]_D^{26}$= +6.5 (H_2O, c = 2.0) (lit.[18] $[\alpha]_D^{19}$ = +7.3, H_2O, c = 2.0, D-enantiomer); Images of ^1H and ^{13}C NMR spectra reported in Section 5; IR (neat): ν 3350, 2923, 2466, 1439, 1702, 1666, 1615, 1439, 1373, 1190, 1135, 1083, 1035, 841, 799, 724, 600 cm^{-1}; HRMS (ESI–): Calculated for $C_6H_9O_6^-$: 177.0394; Found: 177.0404.

5. ^{1}H and ^{13}C NMR spectra of D-KDGlc and L-KDGal

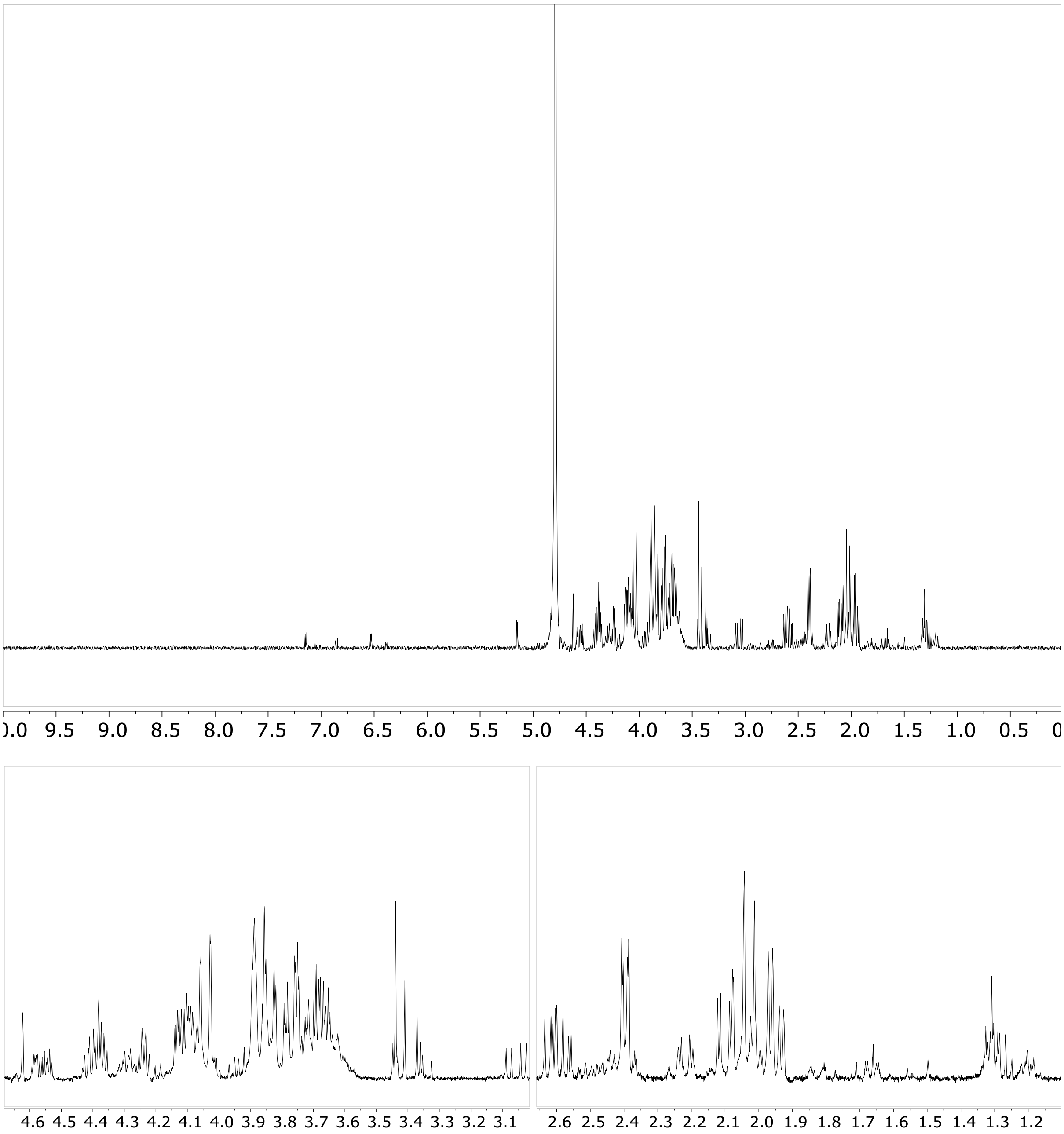

Figure S9. ^{1}H NMR (500 MHz, D_2O) of D-KDGlc.

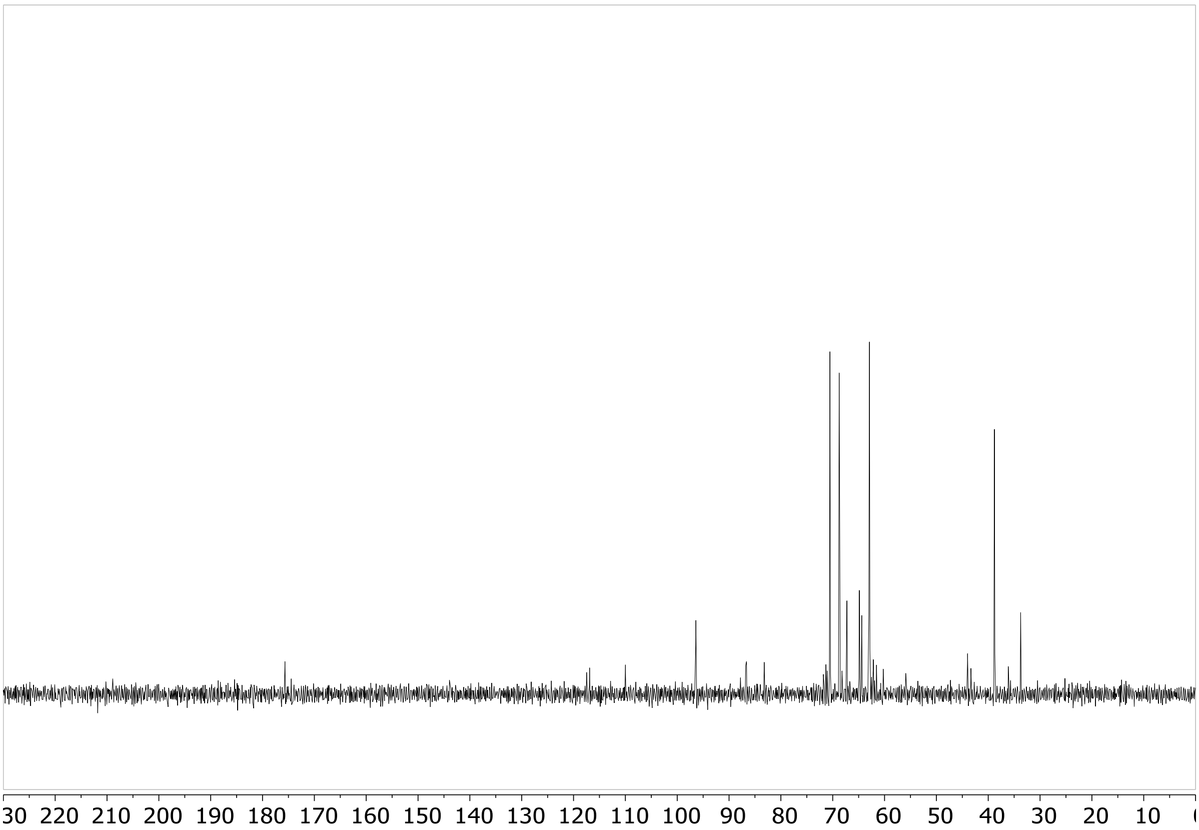

Figure S10. $^{13}C\{^1H\}$ NMR (126 MHz, D_2O) of D-KDGlc.

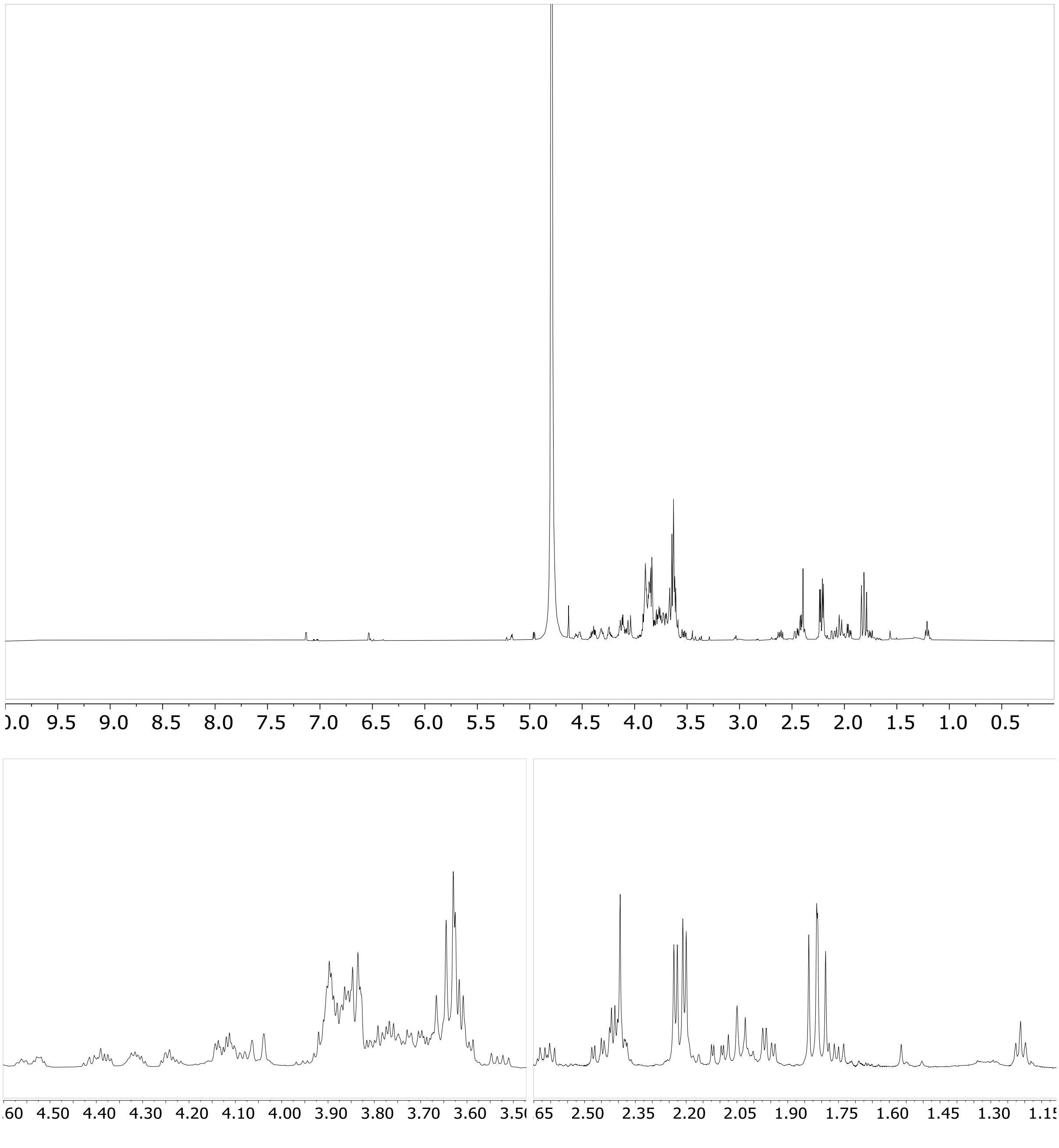

Figure S11. ^{1}H NMR (500 MHz, D_2O) of L-KDGal.

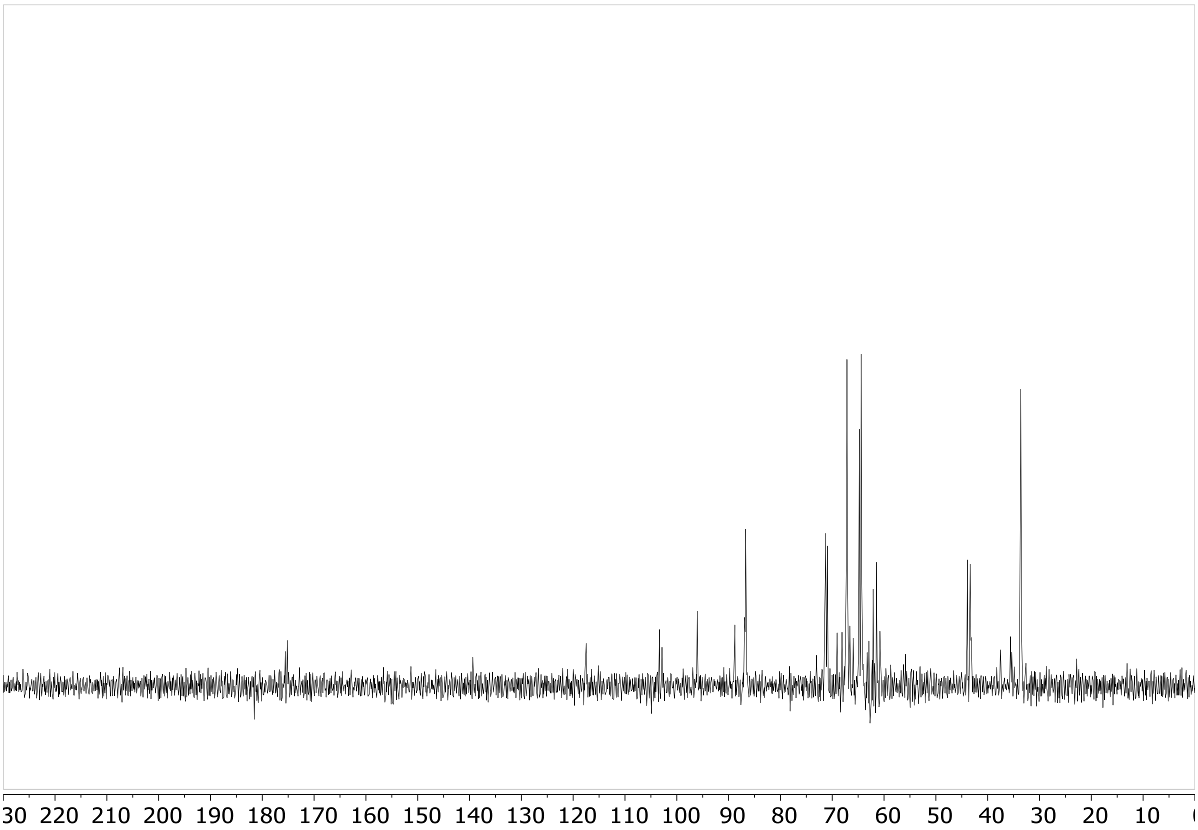

Figure S12. $^{13}C\{^{1}H\}$ NMR (126 MHz, D_2O) of L-KDGal.

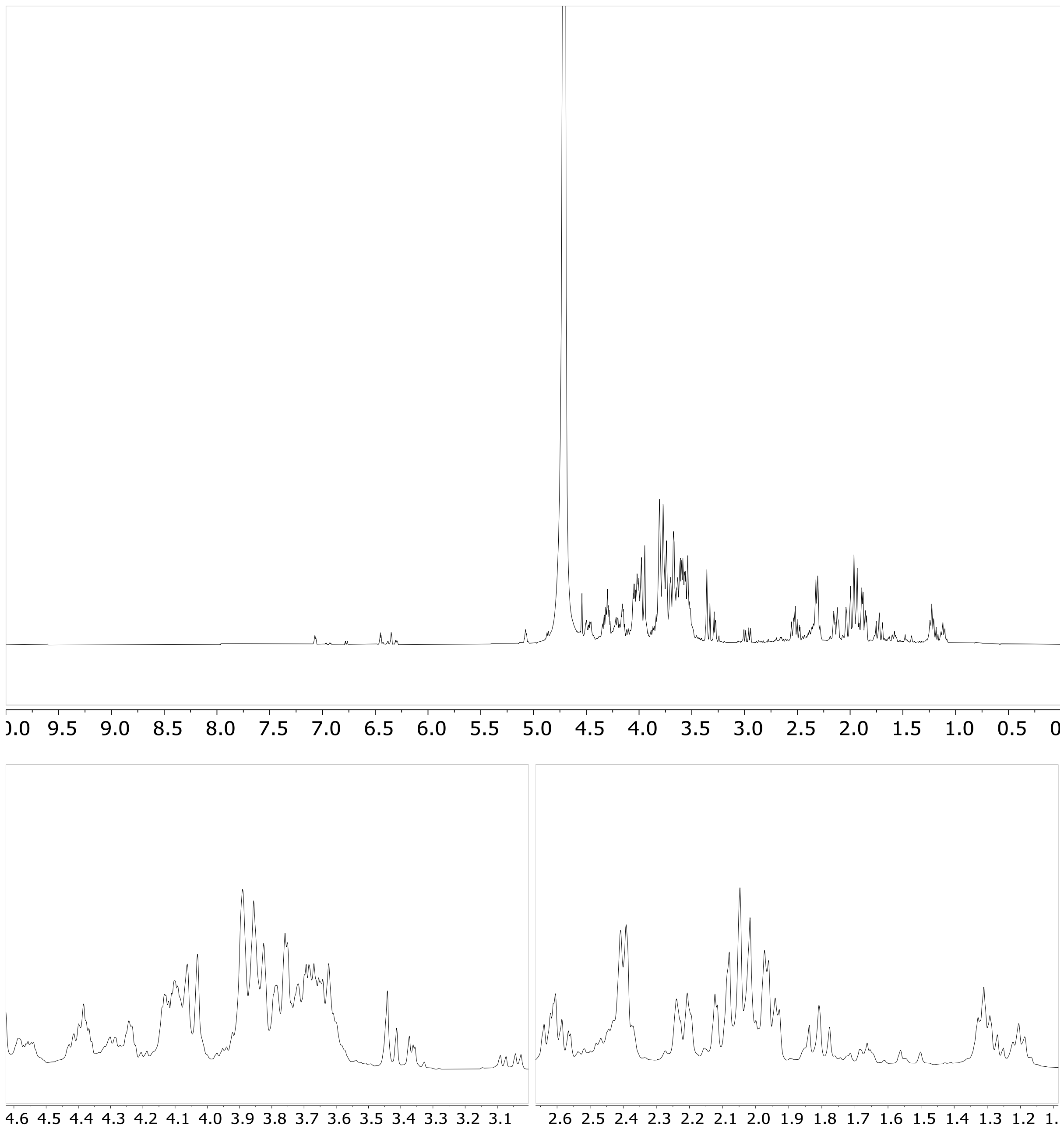

Figure S13. ^{1}H NMR (500 MHz, D_2O) of a 1:1 mixture of D-KDGlc and D-KDGal.

6. References

(1) Royer S.F.; Haslett L.; Crennell S.J.; Hough D.W.; Danson M.J.; Bull, S. D. Structurally informed site-directed mutagenesis of a stereochemically promiscuous aldolase to afford stereochemically complementary biocatalysts. *J. Amer. Chem. Soc.,* 2010*,* **132**, 11753-11758.

(2) Zinovjev, K.; van der Kamp, M. W. Enlighten2: molecular dynamics simulations of protein-ligand systems made accessible. *Bioinformatics (Oxford, England),* 2020. DOI: 10.1093/bioinformatics/btaa643.

(3) *AMBER 2018*; University of California, San Francisco, 2018.

(4) Sondergaard, C. R.; Olsson, M. H. M.; Rostkowski, M.; Jensen, J. H. Improved Treatment of Ligands and Coupling Effects in Empirical Calculation and Rationalization of pK(a) Values. *J. Chem. Theory Comput.* 2011, **7** (7), 2284-2295. DOI: 10.1021/ct200133y.

(5) Olsson, M. H. M.; Sondergaard, C. R.; Rostkowski, M.; Jensen, J. H. PROPKA3: Consistent Treatment of Internal and Surface Residues in Empirical pK(a) Predictions. *J. Chem. Theory Comput.* 2011, **7** (2), 525-537. DOI: 10.1021/ct100578z.

(6) Maier, J. A.; Martinez, C.; Kasavajhala, K.; Wickstrom, L.; Hauser, K. E.; Simmerling, C. ff14SB: Improving the Accuracy of Protein Side Chain and Backbone Parameters from ff99SB. *J. Chem. Theory Comput.* 2015, **11** (8), 3696-3713. DOI: 10.1021/acs.jctc.5b00255.

(7) Wang, J.; Wolf, R. M.; Caldwell, J. W.; Kollman, P. A.; Case, D. A. Development and testing of a general amber force field. *J Comput Chem* 2004, **25** (9), 1157-1174. DOI: 10.1002/jcc.20035.

(8) Roe, D. R.; Cheatham, T. E. PTRAJ and CPPTRAJ: Software for Processing and Analysis of Molecular Dynamics Trajectory Data. *J. Chem. Theory Comput.* 2013, **9** (*7*), 3084-3095. DOI: 10.1021/ct400341p.

(9) Vanquelef, E.; Simon, S.; Marquant, G.; Garcia, E.; Klimerak, G.; Delepine, J. C.; Cieplak, P.; Dupradeau, F. Y., R.E.D. Server: a Web Service for Deriving RESP and ESP Charges and Building Force Field Libraries for New Molecules and Molecular Fragments. *Nucleic Acids Res.* **2011,** *39*, W511-7.

(10) De Alvarenga, E. S.; Carneiro, V. M. T.; Silvério, F. O.; Saliba, W. A. A high yield synthesis of 1,2:5,6-di-*O*-isopropylidene-D-mannitol. *J. Chil. Chem. Soc.*, 2006, **51**, 986–988. DOI: 10.4067/S0717-97072006000300013.

(11) P. Bentler, N. Erdeljac, K. Bussmann, M. Ahlqvist, L. Knerr, K. Bergander, C. G. Daniliuc and R. Gilmour, Stereocontrolled Synthesis of Tetrafluoropentanols: Multivicinal Fluorinated Alkane Units for Drug Discovery, *Org. Lett.*, 2019, **21**, 7741–7745. DOI: 10.1021/acs.orglett.9b02662.

(12) Ferrié, L.; Ciss, I.; Fenneteau, J.; Vallerotto, D.; Seck, M.; Figadère, B. Amphidinolides F and C2: An Odyssey in Total Synthesis. *J. Org. Chem.*, 2022, **87**, 1110–1123. DOI: 10.1021/acs.joc.1c02458.

(13) Ralston, K. J.; Ramstadius, H. C.; Brewster, R. C.; Niblock, H. S.; Hulme, A. N. Self-Assembly of Disorazole C1 through a One-Pot Alkyne Metathesis Homodimerization Strategy. *Angew. Chemie Int. Ed.*, 2015, **54**, 7086–7090. DOI: 10.1002/anie.201501922.

(14) Lamble, H. J.; Danson, M. J.; Hough, D. W.; Bull, S. D. Engineering stereocontrol into an aldolase-catalysed reaction. *Chem. Commun.*, 2005, 124–126. DOI: 10.1055/s-0031-1291136.

(15) Stecko, S.; Michalak, M; Stodulski, M.; Muchał, Ł.; Parda, K.; Furman, B.; Chmielewski, M. Practical One-Pot Synthesis of Protected L-Glyceraldehyde Derivatives. *Synthesis*, 2012, **44**, 2695–2698. DOI: 10.1055/S-0031-1291136.

(16) Chiang, Y.; Kresge, A. J.; Pruszynski, P. Keto-Enol Equilibria in the Pyruvic Acid System: Determination of the Keto-Enol Equilibrium Constants of Pyruvic Acid and Pyruvate Anion and the Acidity Constant of Pyruvate Enol in Aqueous Solution. *J. Am. Chem. Soc.*, 1992, **114**, 3103–3107. DOI: 10.1021/ja00034a053.

(17) Watthaisong, P.; Binlaeh, A.; Jaruwat, A.; Lawan, N.; Tantipisit, J.; Jaroensuk, J.; Chuaboon, L.; Phonbuppha, J.; Tinikul, R.; Chaiyen, P.; Chitnumsub, P.; Maenpuen, S. Catalytic and structural insights into a stereospecific and thermostable Class II aldolase HpaI from Acinetobacter baumannii, *J. Biol. Chem.*, 2021 297, 101280. Doi: 10.1016/j.jbc.2021.101280

(18) Lamble, H. J.; Royer, S. F.; Hough, D. W.; Danson, M. J.; Taylor, G. L.; Bull, S. D. A Thermostable Aldolase for the Synthesis of 3-Deoxy-2-ulosonic Acids. *Adv. Synth. Catal.*, 2007, **349** (6), 817–821. DOI: 10.1002/ADSC.200600520.
